# Comprehensive description of genomewide nucleotide and structural variation in short‐season soya bean

**DOI:** 10.1111/pbi.12825

**Published:** 2017-11-03

**Authors:** Davoud Torkamaneh, Jérôme Laroche, Aurélie Tardivel, Louise O'Donoughue, Elroy Cober, Istvan Rajcan, François Belzile

**Affiliations:** ^1^ Département de Phytologie Université Laval Quebec City QC Canada; ^2^ Institut de Biologie Intégrative et des Systèmes (IBIS) Université Laval Quebec City QC Canada; ^3^ CÉROM Centre de Recherche Sur Les Grains Inc. Saint‐Mathieu de Beloeil QC Canada; ^4^ Agriculture and Agri‐Food Canada Ottawa ON Canada; ^5^ Department of Plant Agriculture, Crop Science Bldg. University of Guelph Guelph ON Canada

**Keywords:** bioinformatics pipeline, genotype accuracy, heterozygosity, next‐generation sequencing, sequence variants, SVs

## Abstract

Next‐generation sequencing (NGS) and bioinformatics tools have greatly facilitated the characterization of nucleotide variation; nonetheless, an exhaustive description of both SNP haplotype diversity and of structural variation remains elusive in most species. In this study, we sequenced a representative set of 102 short‐season soya beans and achieved an extensive coverage of both nucleotide diversity and structural variation (SV). We called close to 5M sequence variants (SNPs, MNPs and indels) and noticed that the number of unique haplotypes had plateaued within this set of germplasm (1.7M tag SNPs). This data set proved highly accurate (98.6%) based on a comparison of called genotypes at loci shared with a SNP array. We used this catalogue of SNPs as a reference panel to impute missing genotypes at untyped loci in data sets derived from lower density genotyping tools (150 K GBS‐derived SNPs/530 samples). After imputation, 96.4% of the missing genotypes imputed in this fashion proved to be accurate. Using a combination of three bioinformatics pipelines, we uncovered ~92 K SVs (deletions, insertions, inversions, duplications, CNVs and translocations) and estimated that over 90% of these were accurate. Finally, we noticed that the duplication of certain genomic regions explained much of the residual heterozygosity at SNP loci in otherwise highly inbred soya bean accessions. This is the first time that a comprehensive description of both SNP haplotype diversity and SV has been achieved within a regionally relevant subset of a major crop.

## Introduction

Genetic variation describes the occurrence of DNA sequence differences among individuals of the same species (Hedrick, 2011). Genetic variation is highly advantageous in an evolutionary sense as it enhances adaptability and survival of a population in the face of changing environmental conditions and other unexpected circumstances (Dobzhansky, 1970; Hedrick, 2011). Genetic variation can be broadly divided into two major categories: nucleotide and structural variations. Nucleotide variants are usually defined as encompassing single or multiple nucleotide variants (SNPs, MNPs) and small insertions/deletions (indels), whereas structural variants (SVs) represent larger rearrangements of various types [deletions, insertions, inversions, translocations, duplications and copy number variations (CNVs)] (Tuzun *et al*., 2005). The advent of next‐generation sequencing (NGS) technologies has provided an exceptional opportunity to systematically detect both nucleotide and structural variants in plant and animal genomes (Church, 2006; El‐Metwally *et al*., 2014; Hall, 2007).

NGS has facilitated greatly the development of methods to genotype very large numbers of nucleotide variants such as single nucleotide polymorphisms (SNPs) (Goodwin *et al*., 2016). In a complementary approach, NGS has been exploited to simultaneously identify and genotype informative SNPs, without the need for any prior knowledge of these polymorphic loci, using complexity reduction approaches such as genotyping‐by‐sequencing (GBS) (Davey *et al*., 2011). Finally, decreased whole‐genome sequencing (WGS) costs have made it possible to sequence entire genomes of numerous individuals, cultivars or accessions of the same species (Gudbjartsson *et al*., 2015; Zhang *et al*., 2001; Zhou *et al*., 2015).

NGS technologies now allow large quantities of high‐quality DNA sequence data to be generated at modest cost (Zhang *et al*., 2001). However, despite considerable advances in algorithm development, the processing of these massive amounts of sequence data into high‐quality variant calls remains challenging (Muir *et al*., 2016). To date, several tools have been developed to discover and genotype nucleotide variants, while SV detection and calling algorithms are relatively recent (Hwang *et al*., 2015). Decoding the raw sequencing data into a catalogue of nucleotide variants and genotype calls requires two essential steps: read mapping and variant/genotype calling. First, reads are aligned against a reference genome, variable sites are identified and genotypes at those sites are determined (Nielsen *et al*., 2011). In addition to calling SNPs and small indels, however, bioinformatics tools have been developed to allow the discovery and genotyping of larger sequence variants (Abyzov *et al*., 2011; Chen *et al*., 2009; Layer *et al*., 2014). To date, three major strategies have been exploited to identify structural variants from aligned reads: depth of coverage, paired‐end mapping and split‐read mapping. Depth of coverage is designed to detect changes in the number of reads that align to a given region in the genome. A reduction or an increase in this coverage can suggest that a deletion or an increase in the copy number of a sequence has occurred in a given individual compared to the reference genome. When paired‐end sequencing is used, it can be assumed that the two sequences that form a pair originate from a single DNA fragment, and thus lie in close proximity on an opposite strand of the reference genome. In the paired‐end mapping approach, when paired reads deviate from this expectation, either because they map to sites that are too far apart or are no longer on opposite strands, this suggests that the individual sample from which these paired reads were generated differed from the reference genome in some structural fashion. Finally, in the case of split reads, this strategy exploits the fact all structural rearrangements generate breakpoints that are analogous to ‘scars’. The ‘scars’ produce sequence reads that contain base pairs that are not contiguous in the reference genome. If two portions of a single sequence read align to different places in the reference genome, this suggests that a rearrangement has occurred (Marroni *et al*., 2014).

To date, the genetic dissection of complex traits in plants and animals has relied almost exclusively on nucleotide variants either as markers of a closely associated mutation or as the direct causal mutation. In recent years, several studies have illustrated the functional impact of SVs in human disease, plant phenotypes and disease resistance (Carvalho and Lupski, 2016; Cook *et al*., 2012). Therefore, no characterization of genetic diversity is complete without the description of both nucleotide and structural variation.

In this study, we describe the WGS of 102 short‐season soya bean accessions [(*G. max* L.), a palaeopolyploid (diploidized tetraploid)] to identify both nucleotide and structural variants using a combination of several bioinformatics tools. We then measure the accuracy of these variants through validation experiments and describe their distribution in the soya bean genome. We also show the impact of joint analysis of nucleotide and structural variants in elucidating the cause of residual heterozygous genotypes observed in inbred lines that are expected to be fixed at all loci.

## Results

### Nucleotide variation

#### Discovery and genotyping

We selected 102 Canadian short‐season elite soya bean accessions for whole‐genome sequencing based on a prior genetic analysis containing a larger set of accessions (*n *= 441) that had been genotyped with ~80 K SNPs using a genotyping‐by‐sequencing (GBS) approach (Figure S1). This collection of 102 samples was selected based on genetic distance to cover genetic diversity of short‐season soya bean germplasm. The accessions were sequenced using Illumina short‐read technology (100‐ or 125‐bp reads) to a median depth of 11× (Table S1). A total of 1.02 × 10^9^ high‐quality trimmed reads (Phred quality score >32) were used to call nucleotide variation in this data set. In total, 93.6% of the reads were successfully mapped to the soya bean reference genome (Williams 82, Schmutz *et al*., 2010). On average, a coverage of at least 1× was achieved for 956 Mb (excluding gaps), thus covering 97.6% of the *G. max* genome sequence.

To date, all variant calling from WGS data in soya bean has been performed using the SOAPsnp pipeline. Prior to conducting large‐scale variant calling on all accessions, however, we first tested the performance and speed of four genotyping pipelines/tools: Fast‐WGS (developed in‐house, see description in Data S1), SOAPsnp, GATK HC and SAMtools on a subset of only 10 accessions. All four called a similar number of SNPs (~1.7 M) and indels (~270 K), but vast differences were observed in terms of the time needed to complete this analysis (23 h, 61 h, 581 h and 238 h, respectively) on the same server (Linux, 48 CPU, 1 Tb RAM). Based on these results, we chose to conduct an analysis on the entire set of accessions only with the two fastest pipelines: Fast‐WGS and SOAPsnp. We then analysed the complete set of reads (for all accessions) with these two pipelines under the same variant‐calling conditions. As shown in Table 1, Fast‐WGS called slightly more (7.2%) total variants due to either base substitutions (SNPs and MNPs) or small indels (4 998 229 vs 4 636 634). Of these, close to 1 M variants were identified as novel polymorphisms not previously recorded in dbSNP among the *Glycine* spp. (Data S2).

**Table 1:** Number of detected variants using two different WGS variant‐calling pipelines (Fast‐WGS and SOAPsnp)

Pipeline/Variants	SNPs	MNPs	Indels	Computing time^a^
Fast‐WGS	4 071 378	284 836	642 015	81 h
SOAPsnp	4 124 216	ND	512 418	261 h

a Analysis was performed using a Linux server with 64 CPU and 1 Tb of RAM.

To assess and compare the quality of genotype calls, we compared our WGS data with the SoySNP50K array data for 19 accessions for which these data were available. Globally, more than 600K genotype calls (35 481 SNP loci × 19 samples) could be compared in this fashion, of which 0.25% were presumed to be indels when no genotype (missing data) was indicated for a given site in a given accession in the SoySNP50K data. As can be seen in Table 2, the quality of the genotype calls made using Fast‐WGS was higher for all three types of genotype calls; the degree of concordance with the calls made on the SoySNP50K array increasing by between 2.6 and 6.8% relative to those observed for the SOAPsnp data. This analysis suggests that a higher level of genotypic accuracy could be obtained for the soya bean SNP data sets currently available using the Fast‐WGS pipeline.

**Table 2:** Accuracy of genotype calls made using two WGS variant‐calling pipelines (Fast‐WGS and SOAPsnp). WGS‐derived SNP genotypes were compared to the genotypes called at loci in common with the SoySNP50K array for the same samples

Variants/Pipeline	Fast‐WGS	Concordance (%)	SOAPsnp	Concordance (%)
Shared genotypes^a^	674 139		645 070	
Homozygous	668 672	99.7	641 215	97.1
Heterozygous	3842	98.6	2152	91.8
Indels	1625	96.1	1703	89.5

a Shared genotypes with the SoySNP50K data set.

The SNP data set obtained using Fast‐WGS contained 9% missing data. We wanted to test how accurately these could be imputed. After imputation of these missing data, we compared the imputed genotypes with the subset of corresponding genotypes obtained using the SoySNP50K array. As can be seen in Table 3, from 600 K shared genotypes (see above), 635 genotypes that were missing in our initial WGS data (while present in SoySNP50K data for the same sample) were imputed and directly compared with their counterparts in the array data. Of these, 41 were heterozygous while the remainder (594) were homozygous. We found a high level of concordance between these two data sets (imputed and SoySNP50K), with 98.8 and 92.7% of homozygous and heterozygous genotypes having been correctly imputed, respectively. Taken together (original calls + imputed calls) across all three types of variants, we found that 99.6% (672 005/674 139) of the genotypes obtained using the Fast‐WGS pipeline (including imputed data) proved to be in agreement with the genotypes obtained at loci in common with the SoySNP50K array.

**Table 3:** Accuracy of imputed missing data in the WGS SNP data set. Imputed genotypes were compared to the genotypes called at loci in common with the SoySNP50K array for the same samples

Variants	WGS data set	Imputation accuracy (%)
Number of homozygous genotypes	594	98.8
Number of heterozygous genotypes	41	92.7
Total	635	98.6

### Variant annotation and prediction of their functional impact

**Figure 1:**
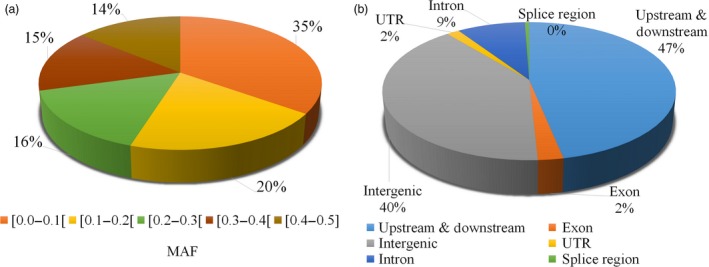
(a) Minor allele frequency (MAF) of variants. (b) Location of variants within the genome.

We grouped sequence variants into five categories based on the observed minor allele frequency (MAF). As can be seen in Figure 1a, 35% of sequence variants were present in up to 10 samples ([0.0–0.1]) and 14% were present at an almost equal frequency with the other allele ([0.4–0.5]). Almost half of these variants were present in the immediate vicinity of genes (up‐/downstream regions (5 kb before and after gene), 47%) or intergenic regions (40%), while exonic and intronic regions contained only 2% and 9% of variants, respectively (Figure 1b). Also splice sites contained very few variants with only 0.1% of the total.

**Figure 2:**
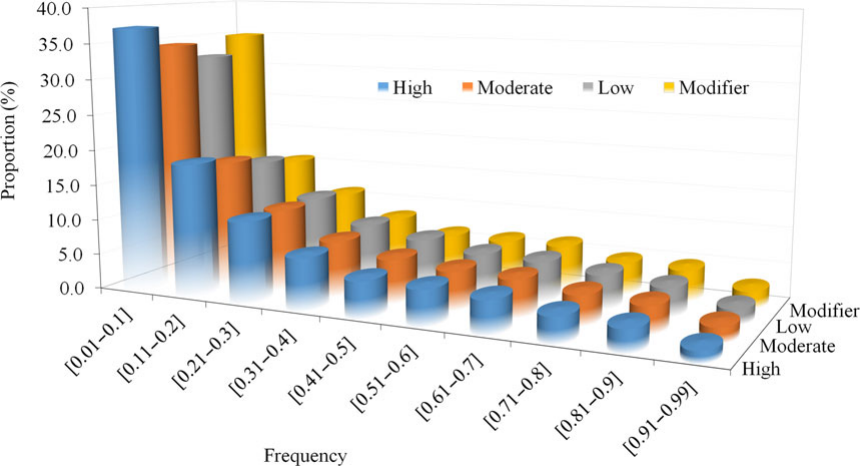
Distribution of variants with different degrees of predicted functional impact based on mutant allele frequency.

We then grouped all observed sequence variants into four categories based on the predicted functional impact of the observed mutation: i) high (0.071%) variants, which are predicted to have a disruptive impact on the protein, probably leading to protein truncation, loss of function or triggering non‐sense‐mediated decay; ii) moderate (1.341%), nondisruptive variants that might change the protein effectiveness (mis‐sense variants and in‐frame deletions); iii) low (1.1%), mostly harmless or unlikely to change protein behaviour (synonymous variants); and iv) modifier (97.48%), noncoding variants. Figure 2 presents the frequency distribution of these four predicted functional impact categories of the mutant (alternative) allele. All four of these categories of mutations showed a similar distribution with most mutations being present at relatively low frequency (<20%) and only a small subset being present at high frequency (>80%).

From a functional standpoint, we were particularly interested in the subset of mutations predicted to have a large impact. Although these represent only a small fraction of all sequence variants (0.071%), this still corresponds to 4113 variants in 3064 genes. Of these variants, 2279 were SNPs, 230 MNPs and 1604 indels. Although only 12% of the sequence variants were indels, they were over‐represented in this category, owing to their tendency to shift the reading frame when they occur in exons. Thus, indels represented 39% of the 4113 functionally high impact variants. In total, we detected 1418 frameshift, 1378 splice receptor/donor, 1251 stop‐gained and 185 start/stop lost variants. As expected, the largest proportion of these variants (35.5%, 1461/4113) were present at a low frequency (<10%). On the other hand, a total of 331 mutations in 238 genes (7.8%) were present in the vast majority of these soya bean lines (frequency ≥0.8) (Figure S2). Owing to the lack of any significant enrichment in terms of GO annotation (data not shown), we investigated the functional annotation of these genes individually using public databases (Table S2). Using the SoyBase and Phytozome databases, we found that of 238 genes, 31 had no annotation nor evidence of expression; we considered these genes as possible pseudogenes. Among the remaining 207 genes, which had annotation and expression profile, we found at least one other functional copy for 177 genes, while the final 30 genes seemed to be unique genes. We suggest that nonsynonymous mutations in these 30 unique genes for which there was evidence of transcriptional activity would be expected to impact plant function significantly in short‐season soya bean. Indeed, *Glyma.10 g221500* (*GmGIa*) (one of these 30 genes) is the gene underlying the maturity locus *E2*. The mutation in exon 10 of this gene is the known causal variant for the *e2* allele (Langewisch *et al*., 2014). As the lines characterized in this work are all adapted to a short growing season, it makes perfect sense that these are fixed for a nonfunctional allele that contributes to earliness.

### Population genetics, LD, haplotypes and untyped genotype imputation

To provide a comprehensive understanding of the population structure among this set of short‐season soya bean lines, we performed three analyses using SNP data: 1) a phylogenetic tree (neighbor‐joining method) with *G. soja* as an outlier; 2) a principal component analysis (PCA); and 3) a STRUCTURE analysis using different K values to detect evidence of admixture in this collection (Figure S3). The neighbor‐joining tree, based on all pairwise genetic distances among the 102 soya bean accessions, showed many distinct branches with *G. soja* as a clear outlier (Figure S3a). Principal component analysis (PCA) also showed that the accessions seemed to form approximately five divergent groups (circled) (Figure S3b). Similarly, using fastSTRUCTURE, the most likely number of subpopulations (K) was five, with most accessions showing some degree of admixture (Figure S3c). The composition of the groups of lines defined using PCA and STRUCTURE was almost identical, with only a few exceptions. This collection of soya bean accessions is composed of lines belonging to different maturity groups (MGs ranging from 000 to I). We tested whether these defined subpopulations could correspond to different MGs, but this did not prove to be the case (data not shown).

**Figure 3:**
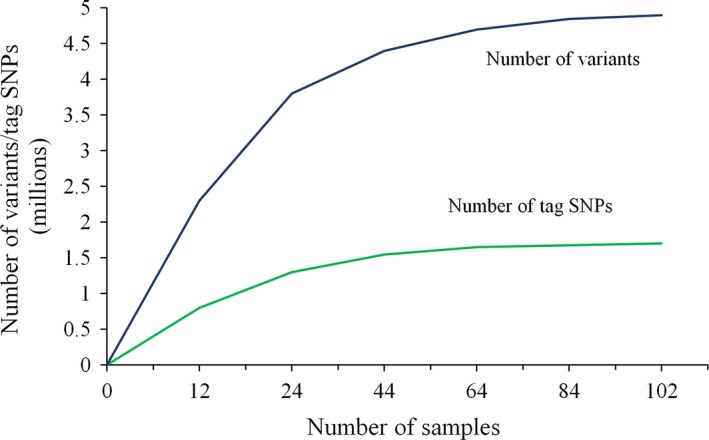
Number of variants (blue) and tag SNPs (green) based on different number of samples.

The extent of linkage disequilibrium (LD) can provide a measure of haplotype diversity in a population. We calculated all pairwise LD (*r* ^2^ and D′) for sequence variants and we found high levels of LD among short‐season soya beans. The average distance over which LD decayed below 0.2 in this population was ~150 kb. Using these LD data, we identified 1.7 million tag SNPs based on haplotypes. To determine if a good level of saturation of both variants and tag SNPs had been achieved among elite short‐season soya bean using this collection of accessions, we analysed randomly selected subsets of samples of increasing size (*N *= 12, 24, 44, 64, 84 and 102). As illustrated in Figure 3, the number of variants discovered did not increase much beyond 80 accessions. Interestingly, the number of tag SNPs reached a plateau much faster; the vast majority of tag SNPs having been discovered within the first set of approximately 40–50 accessions. These results suggest that the current data set offers an exhaustive characterization of the variants and tag SNPs present in the elite Canadian soya bean germplasm.

To test how well this reference panel of variants could serve as a reference panel to impute missing data in data sets derived from lower density genotyping tools, we used a set of ~150 K GBS‐derived SNPs called on a set of 530 short‐season soya bean accessions from Canada. This set of 530 included all 102 accessions characterized by WGS. All tag SNPs that were present in the reference panel but were absent from the GBS‐derived data set (~1.5 M SNPs) were imputed onto the GBS data set. To allow us to estimate the accuracy of this imputation at previously untyped loci, the WGS data from a single accession (among the 102) were left out of the reference panel. Then, the imputed genotypes at untyped loci (not present in the GBS data set) were compared to the actual genotypes revealed through WGS. Five such permutations were done by randomly selecting one accession for removal from the reference panel and imputation. On average, 96.4% of the missing genotypes imputed in this fashion proved to be imputed correctly. As for the 3.6% that were inaccurately imputed, these variants were located in regions with a high degree of haplotype diversity (i.e. low level of LD) and included several rare haplotypes that are difficult to correctly impute. Overall, this data set provides an excellent reference panel for highly accurate imputation of untyped loci in elite short‐season soya bean.

### Structural variation

#### Exploration and characterization

To produce a comprehensive catalogue of large SVs (deletions, duplications, inversions, translocations and CNVs), we used a combination of three bioinformatics tools: LUMPY, BreakDancer and CNVnator. LUMPY using jointly multiple SV signals (read pair, split read and read depth) was able to identify nearly all SV classes except interchromosomal translocations, while BreakDancer (paired‐end SV detection method) was unable to detect small inversions and tandem duplications. CNVnator precisely discover and genotype CNVs (deletions, insertions and duplication) from depth of coverage by mapped reads. Using a combination of different tools allowed us to detect all classes of SVs, and also to do a cross‐validation between outputs of these tools. Among the four types of SVs that were called by three tools (deletions, insertions, inversions and duplications), 91, 87, 86 and 83% of all SVs were called by at least two tools. Thanks to the large predominance and high degree of concordance of deletions and insertions, the mean weighted concordance for these variants reached 89.6%. This result suggests that this catalogue of SVs is highly reproducible using various SV‐calling tools. We produced a unified catalogue of SVs called by at least two of these three bioinformatics tools and these are described in Table 4. This catalogue comprises 63 556 deletions, 16 442 insertions, 2865 duplications, 4221 inversions, 1435 copy number variants and 3313 translocations (intra‐ or interchromosomal). Despite the fact that the size of these SVs spanned a broad range (10 bp to 3 Mb), these rearrangements were typically rather small. Indeed, the median size of the SVs varied between 106 bp (deletions) to 5.6 kb (CNVs). The breakpoints for these SVs could be defined with a variable level of resolution (ranging from 0 to 35 bp) depending on the type of SV. We estimated that deletions, the most abundant type of SV, affected 11.2 Mb (1.1%) of the soya bean genome across all accessions examined. This catalogue of SVs is the first comprehensive characterization and classification of SVs in soya bean and it illustrates the significance of the ‘footprint’ of SVs on the soya bean genome.

**Table 4:** List of structural variant types identified in short‐season soya beans and their characteristics

SV type	Number of SV sites	SV size	Median size of SV (bp)	SV site breakpoint precision (bp)
Deletion	63 556	10 bp–3 Mb	106	±3^a^
Insertion	16 442	32 bp–3 Mb	144	±4^a^
Duplication (disperse duplication)	2865	66 bp–3 Mb	2513	±15^b^
Inversion	4221	33 bp–2.8 Mb	116	±12^c^
CNV (tandem duplication)	1435	500 bp–1.5 Mb	5623	–
Translocation (intrachromosomal)	3011	30 bp–2 Mb	112	±6
Translocation (interchromosomal)	302	100 bp–3 Mb	4523	±35

a Ascertained with split reads.

b Estimated for tandem duplications.

c Estimated for inversions with paired‐end support from both breakpoints.

#### Distribution and annotation of SVs

**Figure 4:**
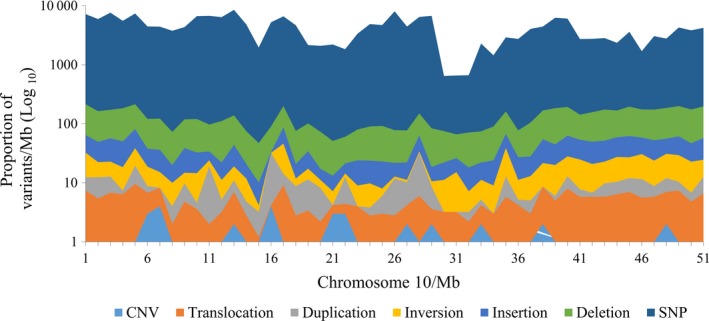
Distribution of SNPs and SVs on chromosome Chr10.

For illustrative purposes, we plotted the distribution of SVs on a single representative soya bean chromosome, Chr 10 (Figure 4). To capture the full range of variant densities (no. of variants/Mb), a logarithmic scale was used. While the most abundant variants were distributed all along the length of this chromosome, CNVs seemed to cluster in certain regions. On the other hand, we saw no correlation between the number of SVs per chromosome and chromosome length (Figure S4). On average, we found that sequence and structural variants are 1.9‐ and 2.3‐fold, respectively, more abundant in euchromatic regions (chromosome arm) than pericentromeric regions. To annotate and identify the potential functional impact of these SVs, we used an in‐house script to identify genes residing within intervals defined by the SV breakpoints (for deletions, duplications and CNVs) or genes in which breakpoints were located (for inversions and translocations) (see M&M and Figure S5 for details). Table 5 shows the number and proportion of the SVs which affected genic regions. In total, 19 424 deletions, 6762 insertions, 2023 duplications, 2286 inversions, 995 CNVs, and 246 translocations impacted genic regions. Overall, 34.5% (31 735/91 832) of SVs were identified as affecting genes and all or almost all of these would be expected to have a strong impact on the function of these genes. Of this number, duplications and CNVs most often affected genic regions (70.6% and 69.3%, respectively), while translocations were the least likely to affect genes (8.2%). These results show that a much higher proportion of SVs are likely to have functional consequences than was the case for smaller variants (SNPs, MNPs and small indels).

**Table 5:** Number of SVs located in genic regions based on their span or breakpoints

SV type	Deletion	Insertion	Duplication^a^	Inversion	CNV^b^	Translocation^c^
In gene	15 365	3201	71	1949	71	164
Upstream and gene	1653	1652	513	147	213	35
Downstream and gene	1714	1579	617	175	267	32
Whole gene	692	329	821	15	443	15
Total	19 424	6762	2023	2286	995	246.6
Percentage of all SVs affecting genes (%)	30.6	41.1	70.6	54.2	69.3	8.2

a Nontandem duplication.

b Tandem duplication.

c Intrachromosomal translocation.

#### Validation of SVs and breakpoints

To estimate the sensitivity and the precision of the results, we selected 40 SVs of different sizes and frequencies within the population for PCR‐based experimental validation. The SVs called on the basis of WGS reads were confirmed by PCR in 80% (32/40) of the cases (Table S3). In all eight cases where we could not confirm a SV by PCR, these were relatively rare, occurring in <7% of the lines. The mean size of these rare and unconfirmed SVs was also much larger than that of the successfully validated SVs (815 kp vs 8 kp). Interestingly, four PCR‐validated SVs were shared by all 102 lines of this collection, suggesting one of three possibilities: 1) these variants are fixed in this particular set of short‐season soya bean, 2) the cultivar used to produce the reference genome (Williams 82) is atypical in its genome structure in these areas, or 3) the reference genome is imperfectly assembled in these regions. We examined the predicted breakpoints defining these SVs by performing Sanger sequencing on PCR amplicons spanning such breakpoints. Sanger sequencing results also confirmed the identified breakpoints at the nucleotide level.

Finally, we sought to examine if we could detect previously described SVs and if these were accurately called in the various accessions. At the *E3* (*GmPhyA3*) locus, some early‐flowering accessions are known to carry the *e3‐tr* allele characterized by a 15.5‐kb deletion that leads to a truncated and nonfunctional phytochrome (Tardivel *et al*., 2014). Similarly, at the *E4* (*GmPhyA2*) locus, many early accessions carry the *e4 (SORE‐1)* allele characterized by the insertion of a 6.2‐kb retroelement (Langewisch *et al*., 2014). In previous work (Tardivel *et al*., 2014), allele‐specific primers had been used to precisely identify the alleles present at these two loci for 50 of the soya bean lines used here and, in all cases, the SVs called on the basis of the WGS reads coincided perfectly with the PCR results (Table S4, Figure S6).

To sum up, we used three different validation strategies: overlap between the SVs discovered by the three tools used, PCR‐based validation and concordance between detected and previously known SVs. Overall, these analyses suggest that the quality of the catalogue of SVs discovered in this study is high.

#### SVs and residual heterozygosity in soya bean

**Figure 5:**
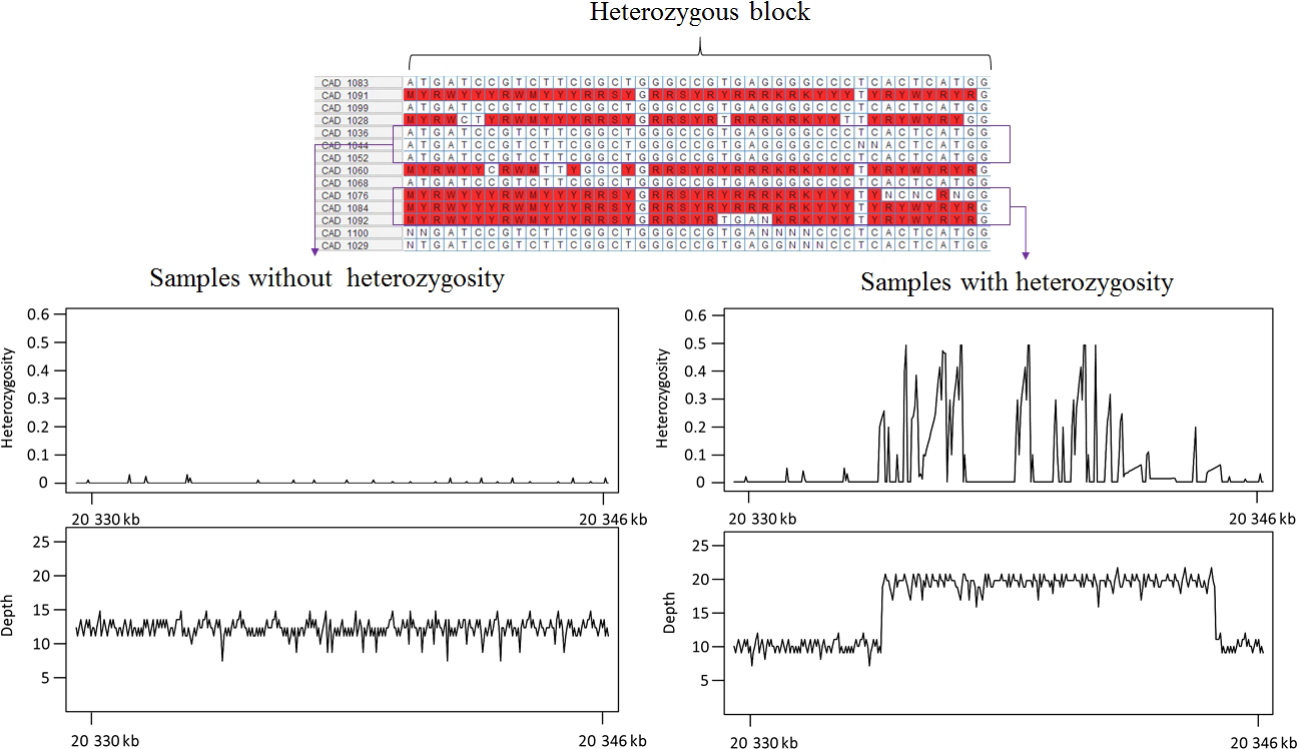
Plot of mapped‐read depth and heterozygosity in a segment of chromosome Chr10 for which some lines exhibited clusters of heterozygous calls, while other lines were homozygous.

Soya bean elite lines are presumed to be highly inbred and, therefore, homozygous. Nonetheless, 3.2% of all genotypes were called heterozygous and, interestingly, a similar proportion was also called as heterozygous using the SoySNP50K array. We wanted to investigate the source of these heterozygous genotypes. Based on their distribution in the genome, these heterozygous genotypes could be qualified as dispersed or clustered. The latter group was almost systematically called heterozygous by both WGS and the array. In contrast, dispersed heterozygous genotypes, although less abundant (~25% of all heterozygous calls), tended not to be in agreement. Therefore, it was possible that some genomic feature could cause both WGS and the array to falsely call heterozygotes. We hypothesized that duplications and CNVs could be involved. As shown in Figure 5a, we saw that in the genomic regions showing a cluster of heterozygous calls, evidence of a duplication or CNV could be found in the form of ‘excess’ read coverage and extended across the same interval affected by heterozygosity. Accessions with the duplicated (or more) genomic segment invariably showed an abnormally high level of heterozygosity, while accessions with a single copy of this segment (as in the reference genome) showed a very low ‘background’ level of heterozygosity (<1%) as seen elsewhere in the genome (Figure 5b). These results show that most residual heterozygosity observed in inbred lines is likely artefactual and the result of duplicated regions leading both the WGS and arrays to make erroneous heterozygous calls. The remaining (dispersed) heterozygous calls (<1% of all called genotypes) are likely a specific artefact of SNP calling based on WGS data.

This observation of a tight link between duplicated regions and the occurrence of heterozygous SNP calls provided us with yet another opportunity to test the validity of our SV calls. We found that 89% of genomic regions that were indicated as being duplicated in specific accessions (based on SV‐calling tools) coincided with regions showing a high level of heterozygosity in the same accessions. This result suggests that close to 90% of the duplications/CNVs called existed in the same set of accessions as those for which heterozygous calls were made.

## Discussion

A first key element to come out of this work is that SVs are a highly important contributor to DNA sequence differences in the soya bean genome. We identified ~5 M nucleotide and only ~92 K SVs among 102 soya bean accessions. At the first glance, there were 54‐fold more nucleotide variants than SVs. In terms of the extent of their ‘fingerprint’ or impact on the genome, however, SVs accounted for a greater proportion of the total nucleotide differences compared to nucleotide variants. Considering only ‘large’ deletions (>10 bp), the former affected more than 1% of the soybean genome compared to **<**0.5% (4.35 M SNPs and MNPs/1.1 Gb) for the nucleotide variants. Thus, the large deletions seem to affect two times more bases compared to all nucleotide variants in the soya bean genome. Similarly, Sudmant *et al*. (2015) demonstrated that, in human genomes, a median of 8.9 Mb of sequence is affected by SVs, compared to 3.6 Mbp for SNPs. This illustrates the importance of characterizing SV, in addition to the nucleotide variants, in the sequenced genomes as these collectively make a very large contribution to the differences that distinguish various accessions within a species.

Beyond the simple quantitative contribution of SNPs and SVs, in terms of nucleotides affected per genome, it is also important to consider the functional impact of these various types of polymorphism. As described in this study (Figure 1 and Table 5), only 2% of nucleotide variants are located in coding regions, and barely 0.071% (4113) were predicted to have a high functional impact. In striking contrast, 34.5% of SVs or their breakpoints (close to 32 k SVs) overlapped completely or partially with genic regions. As a result, a much larger number of genes may be affected functionally by SVs compared to SNPs. Currently, this very significant portion of functionally relevant genomic variation has been, for the most part, ignored in work aiming to identify variants underlying or in close proximity to variants responsible for the phenotypes of interest. Recently, in humans, Sudmant *et al*. (2015) demonstrated that SVs are enriched in haplotypes identified by genomewide association studies and exhibit up to 50‐fold enrichment among expression quantitative trait loci. In addition, these estimates of the impact of SVs on gene function are likely conservative as Lower *et al*. (2009) showed that SVs can affect the expression of genes up to 300 kb away from the variant, whereas the effect of SNPs is generally much more local. We suggest that the collection of SVs identified in this study will help to dissect the genetic basis of important agronomic traits in soya bean.

With the increasing cost‐effectiveness of whole‐genome sequencing projects, the amount of sequence information available to call variants can only increase with time. This requires a constant improvement in the efficiency and speed of SNP‐calling tools to allow for the timely analysis of increasingly large amounts of sequence data. In addition, while many studies have reported on nucleotide variation in soya bean and numerous other species, in our opinion, too little emphasis has been placed on assessing the accuracy of the resulting data. In this study, we used and compared a new bioinformatics analytical pipeline, Fast‐WGS, that is able to efficiently and highly accurately call all three types of nucleotide variants (SNPs, MNPs and indels). In addition to being significantly more rapid (3.2‐fold) than SOAPsnp, it resulted in a significantly more accurate data set, especially with regard to small deletions. In previous studies, lower levels of genotype‐calling accuracy (92–98%) have been reported, and only for SNPs (Hwang *et al*., 2015), whereas using Fast‐WGS achieved similar or higher levels of accuracy for MNPs and indels. We suggest that using Fast‐WGS to process existing WGS data would represent an improvement in the quality and quantity of nucleotide variants available to the research community.

In spite of extensive advancement of sequencing technologies and bioinformatics tools for sequence variant detection, the study of SVs has remained limited to human research (Lam *et al*., 2010; Stankiewicz and Lupski, 2010; Sudmant *et al*., 2015). The main reason for this limitation is the fact that SVs are large‐scale DNA rearrangements that present computational and bioinformatics challenges (Ye *et al*., 2016). We called SVs using a combination of three different tools: LUMPY, BreakDancer and CNVnator. These tools use one or a combination of two to three major referenced‐based mapping approaches (read depth, paired read or split read) to detect SVs (Abyzov *et al*., 2011; Chen *et al*., 2009; Layer *et al*., 2014). It is likely that none of these approaches by itself is sufficient to uncover all SVs (Carvalho and Lupski, 2016). As reported previously, each approach has different strengths and weaknesses in SV detection, which depends on the type of SV or the properties of the underlying sequence at the SV locus (Tattini *et al*., 2015). Using a combination of different tools is important for several reasons: i) algorithms using a split‐read approach can define rearrangement breakpoints, ii) algorithms exploiting read‐depth data have the highest breakpoint resolution for smaller SVs, iii) a paired‐read approach is highly powerful, but lower quality mapping assignments in repetitive regions is challenging and accurate prediction of SV breakpoints depends on very tight fragment size distributions (Quinlan *et al*., 2010). Alkan *et al*. (2011) showed that paired‐read and split‐read methods had the greatest extent of overlap (~67%) in terms of the SVs called, while read‐depth and split‐read approaches were the most discordant, with fewer than 20% of SVs detected by one approach detected by the other. It was found that the main differences in SV detection between these approaches were primarily in duplication‐ and repeat‐rich regions, consistent with what we found in this study. We used these three complementary approaches to overcome the weakness of each approach.

As was done for nucleotide variation, we attempted to assess the reproducibility and the accuracy of SV data set, although this is inherently much more challenging than for nucleotide variation due to the complex and large‐scale nature of many rearrangements and the lack of an independent source of data on structural variation (such as CGH data) for this collection of accessions. A first indication of the quality of the SV data was the observation that close to 90% of all variants were called with more than one tool. In a second approach, we examined if the characteristics of the SVs uncovered in this work were similar to those reported in other species. In terms of the size and type of SVs, we found that 93% were **<**1 kb in size and that 69% of all SVs were deletions. Similarly, Mills *et al*. (2011) sequenced 185 human genomes and created a SV map that encompassed 22 025 deletions and 6000 additional SVs, including insertions and tandem duplications. Furthermore, they reported that more than 90% of the discovered events were **<**1 kb in size and most of these were deletions rather than insertions. In a third approach, somewhat limited in scope, we performed a direct validation on a subset of SVs using PCR and Sanger sequencing. Here, again a high rate of validation was achieved as 80% of the 40 tested SVs were confirmed, with unconfirmed SVs being typically rare events. Finally, we exploited the fact that clusters of residual heterozygosity could be explained by duplication of the corresponding genomic regions to perform a validation of duplications and CNVs. Using heterozygosity as a hallmark of duplicated regions, we found that close to 90% of predicted duplications and CNVs were validated in this fashion.

A final key finding of this work is that the joint study of nucleotide and structural variation can reveal not only biological but also technical complications. A frequent question that has been raised in previous studies on inbred lines or strains was the origin of the small fraction (2–5%) of heterozygous genotypes in genotype data. In this study, we observed that the SVs (particularly duplications and CNVs) are the main reason for artefactual heterozygous genotype calls in soya bean inbred lines. Duplicated regions can diverge and thus generate reads that are almost identical and that convincingly map onto regions that are present in single copy in the reference genome. This apparent diversity at specific positions in these mapped reads is erroneously taken to indicate heterozygosity. We feel it is highly likely that such artefactual heterozygotes will be encountered in many inbred species and even in haploid organisms in which one would not expect to see any heterozygosity.

## Conclusion and future perspectives

We sequenced 102 elite soya bean lines from Canada, the largest collection of elite soya bean germplasm from a defined geographic region to be sequenced to date. This study is groundbreaking for several reasons: i) for the first time, we characterized all classes of structural variants in soya bean; ii) we have presented a new analytical pipeline (Fast‐WGS) that can facilitate and improve SNP calling using WGS data; iii) the SNP haplotype collection shown in this study can be used as a reference panel to accurately impute missing genotypes at untyped loci in short‐season soya bean (the first such reference panel in soya bean); iv) we have found an explanation for the residual heterozygosity at SNP loci; v) this resource combining both nucleotide and structural variants will help investigate phenotype–genotype associations in a more complete fashion in soya bean.

## Experimental procedures

### Soya bean accessions

In this study, we used three collections of soya bean samples. A first panel of 441 accessions (cultivars/advanced breeding lines) were subjected to genotyping‐by‐sequencing (GBS; *Ape*KI protocol) (Elshire *et al*., 2011; Sonah *et al*., 2013), and SNPs were called using the Fast‐GBS pipeline (Torkamaneh *et al*., 2017a). Based on a cladogram produced using these data, a second panel comprising 102 elite accessions (Table S1) were selected to capture the diversity among this collection of short‐season soya bean and were used for WGS (Figure S1). Finally, a set of 89 accessions (mostly advanced breeding lines harbouring traits of interest) was genotyped by GBS, as described above, and added to the collection of 441 accessions to produce a third panel totalling 530 soya bean accessions on which we tested the accuracy of imputation at untyped loci (see below for details).

### Whole‐genome sequencing

Illumina paired‐end libraries were constructed for 102 elite accessions (panel 2 described above) using the KAPA Hyper Prep Kit (Kapa Biosystems, Wilmington, MA, USA) following the manufacturer's instructions (KR0961 – v5.16). Samples were sequenced using the Illumina HiSeq 2500 platform at the Centre Hospitalier de l'Université Laval (CHUL) in Quebec, QC, Canada.

### Choice of WGS analytical pipeline

Two SNP‐calling pipelines were used: SOAPsnp (Li *et al*., 2009) and Fast‐WGS, a new pipeline that we have developed (see details in Data S1). The reads were mapped against *G. max* reference genome [*Gmax*_275 (Wm82.a2.v1)] (Schmutz *et al*., 2010). Every effort was made to call SNPs under comparable conditions. We removed variants if: 1) they had more than two alleles, 2) an allele was not supported by reads on both strands, 3) the overall quality (QUAL) score was <32, 4) the mapping quality (MQ) score was <20, 5) read depth (minNR) was <2 and 6) the minor allele frequency (MinMAF) was <0.02. The final variant catalogue was prepared using Fast‐WGS. Then, we downloaded the catalogue of sequence variants of *Glycine* spp. from dbSNP (build 147), to compare and identify the novel variants detected in this study.

### Genotype accuracy

The SoySNP50K iSelect BeadChip has been used to genotype the entire USDA Soybean Germplasm Collection (Song *et al*., 2015). The complete data set for 19 652 *G. max* and *G. soja* accessions genotyped with 42 508 SNPs was downloaded from Soybase (Grant *et al*., 2010). Of these accessions, 19 were in common with the collection of 102 short‐season soya bean lines characterized here via WGS. For these 19 accessions, we extracted their genotype calls at all SNP loci for which data were available. This large set of SoySNP50K genotype calls (>600 K) was directly compared with the WGS‐derived SNP calls (obtained using one or the other pipeline) using an in‐house script.

### Imputation

To impute missing data in the WGS data set, we used BEAGLE v5 (Browning and Browning, 2007) with the parameters described in Torkamaneh and Belzile (2015). Imputed genotypes at loci in common with the SoySNP50K array were directly compared to those called using the chip. The WGS SNP data from 101 of the 102 resequenced lines were also used as a reference panel to impute missing data onto a collection of 530 accessions (panel 3) previously genotyped with ~150 K GBS‐derived SNPs. The remaining line was kept out of the reference panel to determine how accurately data at untyped loci (present in the WGS data but absent from the GBS catalogue) could be imputed in this line. We performed five such permutations where a single line was kept aside to estimate imputation accuracy. For these lines purposely excluded from the reference panel, we compared the imputed genotypes against the genotypes called at these same loci following WGS.

### Population genetics, LD and tag SNP selection

Population structure was estimated using the Bayesian inference implemented in fastSTRUCTURE (Raj *et al*., 2014). Five runs were performed for each number of populations (K) set from 1 to 12. The most likely K value was determined by the log probability of the data (LnP(D)) and delta K, based on the rate of change in LnP(D) between successive K values. A neighbor‐joining tree was built using MEGA6 (Tamura *et al*., 2013) with 100 bootstraps. Principal component analysis (PCA) was performed using TASSEL v5 and GAPIT (Bradbury *et al*., 2007; Lipka *et al*., 2012) in three dimensions. For tag SNP selection, we used PLINK (Purcell *et al*., 2007) to calculate linkage disequilibrium (LD) between each pair of SNPs within a sliding window of 50 SNPs and we removed all but one SNP that were in perfect LD (LD = 1); the remaining SNPs were deemed tag SNPs.

### Annotation and GO analysis

Functional annotation of nucleotide variation was done by SnpEFF and SnpSift (Cingolani *et al*., 2012) using *G*. *max* reference genome [*Gmax*_275 (Wm82.a2.v1)] (Schmutz *et al*., 2010). Genes containing variants predicted to have a large functional impact were selected from the annotation file. To obtain the description of these genes, we used Phytozome (Goodstein *et al*., 2012) and SoyBase (Grant *et al*., 2010). For gene ontology (GO) analysis, we used the singular enrichment analysis (SEA) method implemented in agri‐GO (Zhou *et al*., 2010).

### Structural variant calling and genotyping

To discover a comprehensive catalogue of SVs from WGS data, we used three tools: LUMPY (Layer *et al*., 2014), BreakDancer (Chen *et al*., 2009) and CNVnator (Abyzov *et al*., 2011). We used SVtyper (Chiang *et al*., 2015) and svtools (Larson *et al*., 2016) for calling the presence or absence of SVs in individual accessions. The raw calls were filtered for 1) the estimated read‐depth ratio (<0.75), 2) the number of spanning read pairs (>10), 3) regions around centromeres (±1 Kb) and 4) regions around assembly gaps (±50 bp). The read‐depth (RD) ratio was calculated as the average RD of the samples that supported the SV divided by the average RD of the samples that did not support the SV. The site list was prepared using an 80% reciprocal overlap (RO) threshold, a maximum breakpoint offset of 250 bp and a genotype quality (phred scaled) >30. Inversions were filtered such that the minimum ratio of genotyped to ungenotyped samples was >0.4, and the fraction of inversions supporting pairs in carriers was >0.3. The translocation calls located in syntenic regions were removed.

### Annotation of structural variants

Functional annotation of SVs was done using an in‐house Python script. We used the *G. max* v2 annotation file to create a genic reference panel in which we recorded the genomic region spanned by each gene. Similarly, we created a file for each SV in which the positions of both breakpoints (start and end) were noted. To detect SVs that had a likely functional impact on genes, we proposed four possible scenarios: (1) a SV was located inside a gene, (2) a SV began in an intergenic region (upstream) and terminated in a gene, (3) a SV began in a gene and terminated in an intergenic region (downstream) and (4) a SV encompassed the gene completely (Figure S5). Using this program, we compared the intervals spanned by SVs with genic intervals to identify partial or complete overlaps.

### Validation of structural variants

We selected two known SVs in known maturity genes (*E3* and *E4*) and 38 random SVs with a focus on translocations and inversions for a PCR‐based validation. Primers were designed using Primer3Plus (Untergasser *et al*., 2007), and their specificity was examined using BLAST on the NCBI and SoyBase databases (Tables S5). Williams82 was used as the reference (control) for PCR. For estimation of breakpoint precision, the PCR products were sequenced using Sanger sequencing.

#### Availability of data and material

The data set supporting the conclusions of this article is available in the NCBI Sequence Read Archive (SRA) repository with the SRP# Study accession, SRP094720 [https://www.ncbi.nih.gov/sra/?term=SRP094720].

Project name: Fast‐WGS.

Project home page: https://bitbucket.org/jerlar73/fast-wgs.

Operating system: Linux.

Programming language: Bash and Python.

License: GNU GPL v3.

Any restrictions to use by nonacademics: No.

## Authors’ contributions

DT, JL, and FB conceived the project. JL and DT contributed to programming. LO, EC and IR contributed to sample selection. AT evaluated SV validation by PCR. DT and FB contributed to writing the manuscript.

## Supporting information


**Data S1** Description of Fast‐WGS. Bioinformatics analytical pipeline for whole‐genome sequencing analysis.
**Data S2** Significant contribution to the public SNP dataset (dbSNP) for *Glycine* spp.
**Figure S1** Cladogram of 441 short‐season soybean accessions from Canada produced using a set of close to 80k SNP markers. Arrows indicate the samples selected for whole‐genome sequencing.
**Figure S2** Distribution of allele frequency for sequence variants located in coding regions and predicted to have a high impact on gene function
**Figure S3** Population genetics analysis.
**Figure S4** Correlation between number of SVs and chromosome length. Deletions (DEL), insertions (INS), copy‐number variations (CNV), duplications (DUP), inversions (INV), and translocations (TRANS).
**Figure S5** Different cases used to identify structural variants that could directly impact the function of a gene.
**Figure S6** Visualized example of PCR‐based genotyping of 10 samples for *E4* gene. *E4* is the wild type form and *e4* resides an insertion.
**Table S1** Information of sequenced short‐season soybean accessions with name and number of trimmed reads (Phred score >32).
**Table S2** List of genes containing variants predicted to have a high impact on gene function.
**Table S3** PCR‐based validation of SVs called on the basis WGS data.
**Table S4** Concordance of WGS‐based genotyping and PCR‐based genotyping results for a deletion in *E3* gene and an insertion in *E4* gene.
**Table S5** Primers used for PCR‐based SV validation.
