# Supplementary material for: Comprehensive description of genomewide nucleotide and structural variation in short‐season soya bean
Source: Plant Biotechnol J. 2017 Nov 3;16(3):749–59. doi: 10.1111/pbi.12825 (PMC5814582; doi:10.1111/pbi.12825)
Supplement: Supplementary file 1 — Data S1 Description of Fast‐WGS. Bioinformatics analytical pipeline for whole‐genome sequencing analysis. Data S2 Significant contribution to the public SNP dataset (dbSNP) for Glycine spp. Figure S1 Cladogram of 441 short‐season soybean accessions from Canada produced using a set of close to 80k SNP markers. Arrows indicate the samples selected for whole‐genome sequencing. Figure S2 Distribution of allele frequency for sequence variants located in coding regions and predicted to have a high impact on gene function Figure S3 Population genetics analysis. Figure S4 Correlation between number of SVs and chromosome length. Deletions (DEL), insertions (INS), copy‐number variations (CNV), duplications (DUP), inversions (INV), and translocations (TRANS). Figure S5 Different cases used to identify structural variants that could directly impact the function of a gene. Figure S6 Visualized example of PCR‐based genotyping of 10 samples for E4 gene. E4 is the wild type form and e4 resides an insertion. Table S1 Information of sequenced short‐season soybean accessions with name and number of trimmed reads (Phred score >32). Table S2 List of genes containing variants predicted to have a high impact on gene function. Table S3 PCR‐based validation of SVs called on the basis WGS data. Table S4 Concordance of WGS‐based genotyping and PCR‐based genotyping results for a deletion in E3 gene and an insertion in E4 gene. Table S5 Primers used for PCR‐based SV validation. [file PBI-16-749-s001.docx]

**Comprehensive Description of Genome-Wide Nucleotide and Structural Variation in Short-Season Soybean**

Davoud Torkamaneh^1,2^, Jérôme Laroche^2^, Aurélie Tardivel^1,2,3^, Louise O’Donoughue^3^, Elroy Cober^4^, Istvan Rajcan^5^ and François Belzile^1,2*^

^1^Département de Phytologie, Université Laval, Quebec City, QC, Canada

^2^Institut de Biologie Intégrative et des Systèmes (IBIS), Université Laval, Quebec City, QC, Canada

^3^CÉROM, Centre de recherche sur les grains inc., Saint-Mathieu de Beloeil, QC, Canada

^4^Agriculture and Agri-Food Canada, Ottawa, ON, Canada

^5^Department of Plant Agriculture, Crop Science Bldg., University of Guelph, Guelph, ON, Canada

* Corresponding author: François Belzile. Tel: 1-418-656-2131#5763; Fax: 1-418-656-7176; Email: francois.belzile@fsaa.ulaval.ca

# **Supplementary data**

## **Supplementary Text**

**Supplementary Text 1.** Description of Fast-WGS. Bioinformatics analytical pipeline for whole-genome sequencing analysis.

**Supplementary Text 2.** Significant contribution to the public SNP dataset (dbSNP) for *Glycine* spp*.*

## **Supplementary Figures**

**Supplementary Figure 1.** Cladogram of 441 short-season soybean accessions from Canada produced using a set of close to 80k SNP markers. Arrows indicate the samples selected for whole-genome sequencing.

**Supplementary Figure 2.** Distribution of allele frequency for sequence variants located in coding regions and predicted to have a high impact on gene function.

**Supplementary Figure 3.** Population genetics analysis. **a)** Phylogenetic tree using Neighbour Joining method, a *Glycine soja* line’s used as outlier. b) Population STRUCTURE analysis using WGS SNPs dataset, representing the existence of five sub-populations in this collection. c) Principal component analysis (PCA) also represented five sub-groups (circled) which are correlated by five sub-population derived from STRACTURE analysis.

**Supplementary Figure 4.** Correlation between number of SVs and chromosome length. Deletions (DEL), insertions (INS), copy-number variations (CNV), duplications (DUP), inversions (INV), and translocations (TRANS).

**Supplementary Figure 5.** Different cases used to identify structural variants that could directly impact the function of a gene. (1) the SV resides entirely within a gene, (2 and 3) a SV encompasses at least part of a gene or one of its breakpoints lies within a gene (4) the SV completely encompasses a gene.

**Supplementary Figure 6.** Visualized example of PCR-based genotyping of 10 samples for *E4* gene. *E4* is the wild type form and *e4* resides an insertion. These results also confirmed the WGS SV genotypes dataset.

## **Supplementary Tables**

**Supplementary Table 1.** Information of sequenced short-season soybean accessions with name and number of trimmed reads (Phred score >32).

**Supplementary Table 2.** Primers used for PCR-based SV validation.

**Supplementary Table 3.** List of genes containing variants predicted to have a high impact on gene function.

**Supplementary Table 4.** PCR-based validation of SVs called on the basis WGS data.

**Supplementary Table 5.** Concordance of WGS-based genotyping and PCR-based genotyping results for a deletion in *E3* gene and an insertion in *E4* gene.

**Supplementary Table 6**. Comparison of the contributions of this and previous large-scale studies to the characterization of both nucleotide and structural variation in soybean.

**Supplementary Text**

**Supplementary Text 1.** Description of Fast-WGS. Bioinformatics analytical pipeline for whole-genome sequencing analysis.

# **Fast-WGS**

A bioinformatics pipeline for processing whole-genome sequencing (WGS) data in view of calling nucleotide variants and small indels.

## **Introduction**

Fast-WGS is a bash pipeline facilitating the processing of FASTQ sequence files obtained by whole-genome sequencing (WGS). It includes a set of bash commands, Python scripts, and well known bioinformatics software such as BWA, SAMtools and Platypus. Users simply provide key information in a parameter file and then launch the program. It requires the existence of a reference genome.

## **Glossary**

This section should allow the user to better understand the terms used throughout the file preparation protocol.

FLOWCELL: Sequencing batch. Originally can contain up to 8 lanes

LANES: File containing multiplexed sequences. Several lanes can be associated to a flowcell.

TECHNOLOGY: Sequencing technology, either Illumina or Ion Torrent

## **Using fastwgs**

The main steps in using Fast-WGS are:

- Run the script ./make_directories.sh that will create the following directories:
  refgenome
  data 
  results
- Prepare the reference genome
- Prepare the data
- Prepare the parameters file
- Run the script fastwgs.sh

## **Dependencies**

In order to use Fast-WGS, you will need the following tools:

- Linux with parallel installed (<http://www.gnu.org/software/parallel/>)
- Python 2.7 or higher (<https://www.python.org/>)
- BWA (<https://github.com/lh3/bwa>)
- SAMtools (<http://www.htslib.org/>)
- Platypus (<http://www.well.ox.ac.uk/platypus>)
- vcf python module (<https://github.com/jamescasbon/PyVCF>)
- fastwgs.sh (this distribution)
- fastwgs_parameters.txt (this distribution)
- make_directories.sh (this distribution)
- vcf2txt.py (this distribution)

## **Additionnal softwares**

This software is not directly in the pipeline but it is recommended for quality check:
- FastQC (<http://www.bioinformatics.babraham.ac.uk/projects/fastqc/>)

## **Preparation the reference genome**

Move your reference genome file in the refgenome directory, move to that directory and index it with the command:
bwa index -a bwtsw refgenome.fasta

The .fai file isn't created as part of "bwa index." To create it, run the command:
samtools faidx refgenome.fasta

That will create a file named refgenome.fasta.fai.

Finally, write the reference genome file name in the parameter file:

REFGEN=refgenome.fasta

## **Preparation the data**

Move your sequence files in the data directory.
For WGS, we use paired-end sequence files and the samples are already demultiplexed. Rename your sample files in the following way:

HN104_1.fq.gz

HN104_2.fq.gz

HN105_1.fq.gz

HN105_2.fq.gz

HN106_1.fq.gz

HN106_2.fq.gz

HN107_1.fq.gz

HN107_2.fq.gz

HN108_1.fq.gz

HN108_2.fq.gz

**It is important to keep the extension .fq.gz because for simplicity it is hardcoded in the pipeline.**

## **Preparation the parameter file**

Verify the information in the file fastwgs_parameters.txt. If necessary, change the value given to the variables. **It is very important not to change the words in capital letters (before the sign =). If you do this, you will get an error message because the pipeline will not find that variable.**
Respect what is an integer and a string.

## **Running Fast-WGS**

To run the pipeline, just enter the command: ./fastwgs.sh fastwgs_parameters.txt

## **Supplementary information**

The parameter file allows the user to change the value of certain variables in the pipeline. However, the list of variables in the parameter file is far from exhaustive. It is therefore important to consider that several parameters of the software used in this pipeline, are hard-coded. We made this choice to make life easier for less experienced users. In line with this philosophy, when the user chooses the ILLUMINA technology in the parameter file, values are automatically given to the following variables in Platypus:

--genIndels=1

--minMapQual=20

--minBaseQual=20

When the IONTORRENT technology is chosen:

--genIndels=0

--minMapQual=10

--minBaseQual=10

These Platypus options are hardcoded in the pipeline:

--maxHaplotypes: 70

--originalMaxHaplotypes: 70

--minFlank: 3

--maxVariants: 10

--badReadsWindow: 5

--largeWindows: 1

--longHaps: 1

--filterReadPairsWithSmallInserts: 0

--minVarFreq: 0.002

--assembleBrokenPairs: 1

However, advanced users can easily edit the fastgbs.sh code and to put the values of their choice.

## **Platypus**

A common problem that can occur with Platypus is where the system says it cannot find a file (BAM) in the sample list to use. This problem is caused by the variable that defines the number of files that can be opened simultaneously. To get the current value used by the system, run the command ulimit -a. By default, this value is usually set to 1024.
To avoid this being limiting, we must consider the number of CPUs you provide to Platypus and the number of samples (bam files) you want to analyze. The number of files that must be opened simultaneously is: # of CPU x # of samples.
The number of CPUs determines the number of genomic regions analyzed simultaneously. For each analyzed genomic region, all bam files must be opened by the program. For example, if you have 180 samples and you give 10 CPUs, this is 1800 files to be opened simultaneously. If the limit of your OS is 1024, you will get an error message. In this case you can give 1024/180 = 5.6, so 5 CPUs. If you can raise the limit 2048 (ulimit -n 2048) you can give 2048/180 = 11.4, 11 CPUs.
To change the limit, enter this command:
ulimit -S -n 5120

## **Quality control**

We strongly recommend users to check for sequence quality with fastqc software.**License**

FastWGS is licensed under the GNU General Public Licence version 3 (GPL3). See the LICENCE file for more details.

**Supplementary Text 2.** Significant contribution to the public SNP dataset (dbSNP) for *Glycine* spp*.*

The Single Nucleotide Polymorphism Database (dbSNP) contains around 16M SNPs and indels for *G. max* and *G. soja*. In total, we identified close to 5M high-quality sequence variants across these 102 short-season elite soybean accessions (*G. max)*. Of these, ~4M (~80%) matched entries in dbSNP (build 147), while close to 1M variants (524,372 SNPs, 284,836 MNPs and 179,116 indels) were identified as novel polymorphisms not previously recorded in dbSNP among the *Glycine* spp. (**Table 1**). Thus, around 20% of the variants identified in the short-season elite germplasm were novel. In terms of the contribution made to dbSNP, while modest in terms of novel SNPs (3.6%), these were much more substantial for indels (13.1%) and especially MNPs (100%).

**Table 1.** Sequence variants detected in Canadian short-season soybean accessions. The number and proportion of variants uncovered in this work that were either present or novel in dbSNP is shown. In addition, the magnitude of the contribution of these new variants to dbSNP is also provided.

| Type | SNPs | | MNPs | | Indels | |
| --- | --- | --- | --- | --- | --- | --- |
| dbSNP | Known | Novel | Known | Novel | Known | Novel |
| Number of variants | 3,547,006 | 524,372 | ND | 284,836 | 462,899 | 179,116 |
| (% of total) | 87.1 | 12.9 | - | 100 | 72.1 | 27.9 |
| Contribution of novel variants to dbSNP (%) | 3.6 | | 100 | | 13.1 | |

**Supplementary Figures**

**Supplementary Figure 1.** Cladogram of 441 short-season soybean accessions from Canada produced using a set of close to 80k SNP markers. Arrows indicate the samples selected for whole-genome sequencing.

**
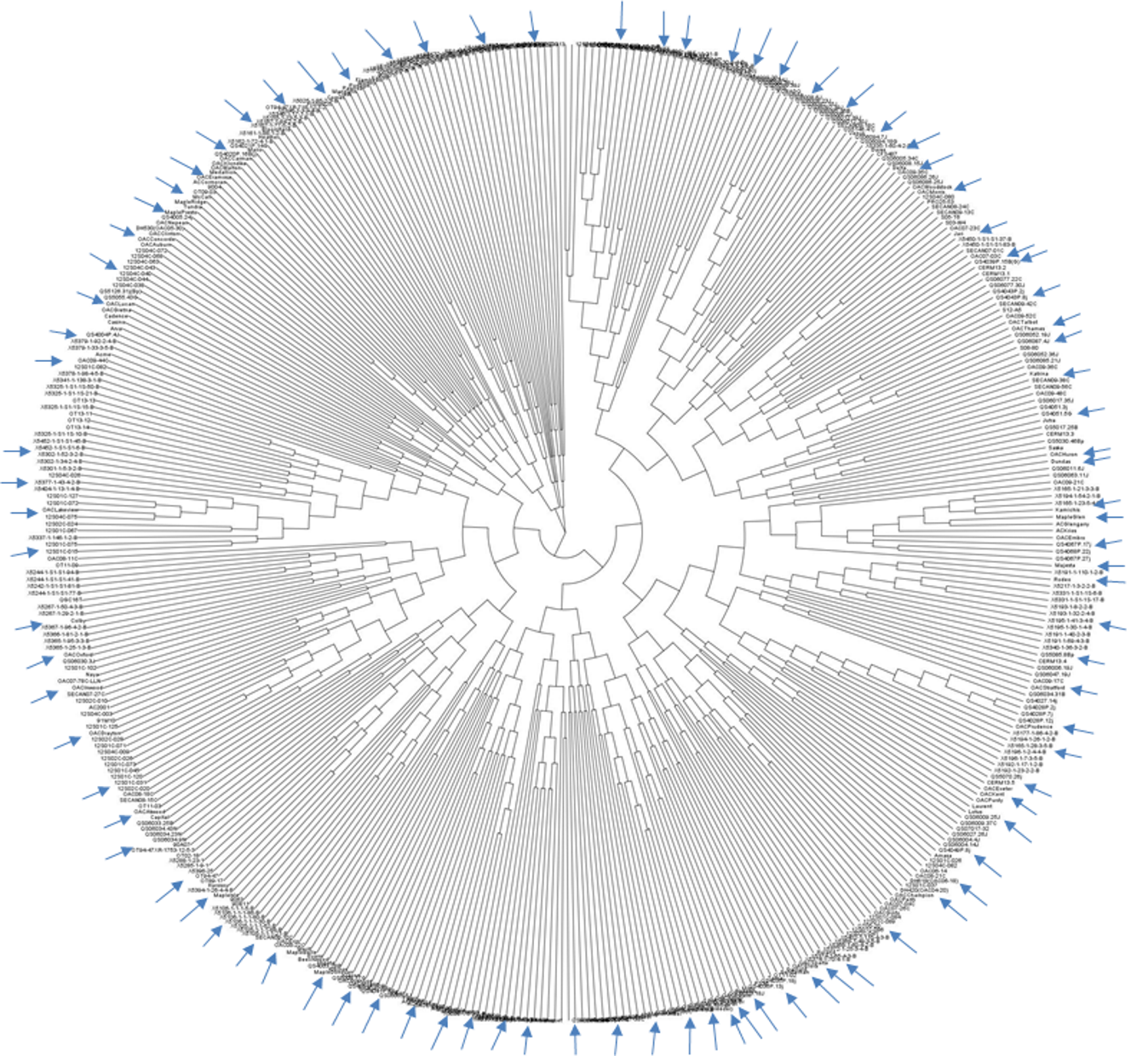
**

**Supplementary Figure 2.** Distribution of allele frequency for sequence variants located in coding regions and predicted to have a high impact on gene function.

**
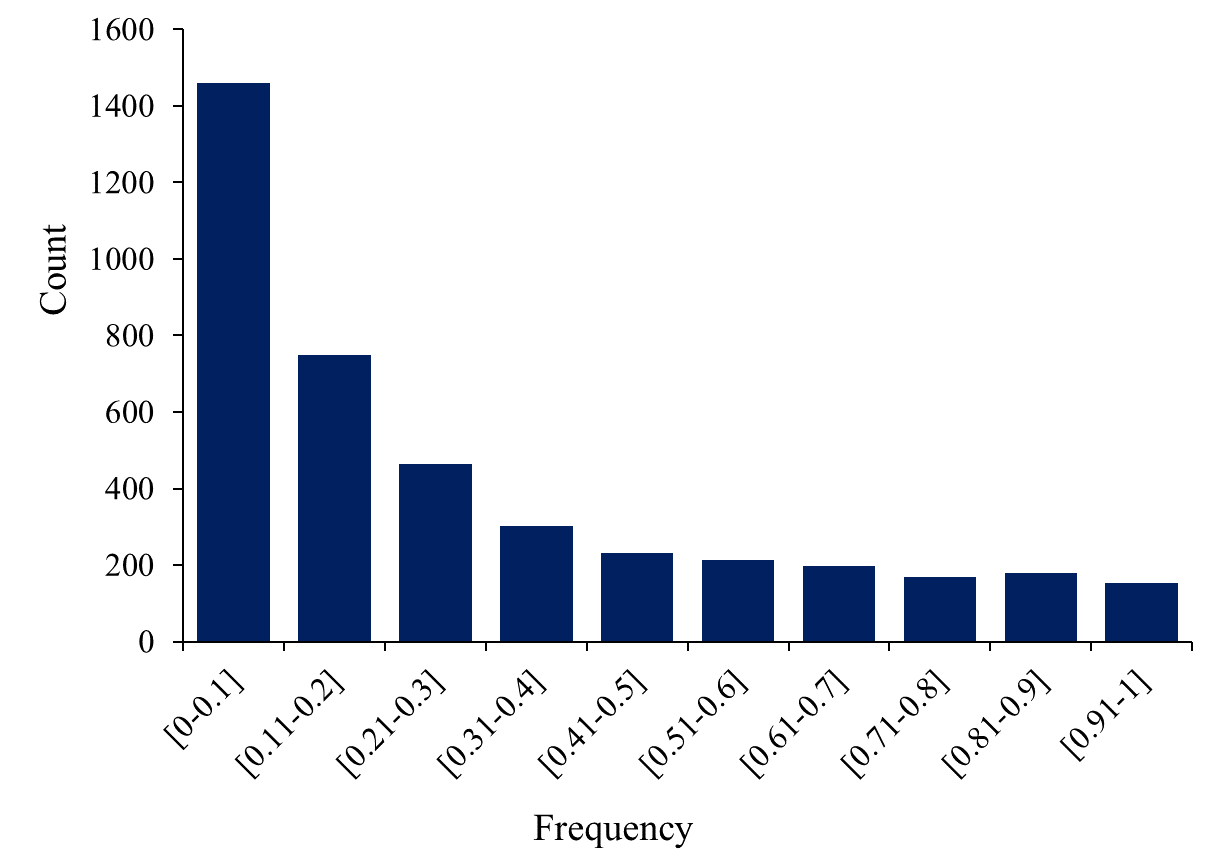
**

**Supplementary Figure 3.** Population genetics analysis. **a)** Phylogenetic tree using Neighbour Joining method, a *Glycine soja* line’s used as outlier. b) Population STRUCTURE analysis using WGS SNPs dataset, representing the existence of five sub-populations in this collection. c) Principal component analysis (PCA) also represented five sub-groups (circled) which are correlated by five sub-population derived from STRACTURE analysis.

**Supplementary Figure 4.** Correlation between number of SVs and chromosome length. Deletions (DEL), insertions (INS), copy-number variations (CNV), duplications (DUP), inversions (INV), and translocations (TRANS).


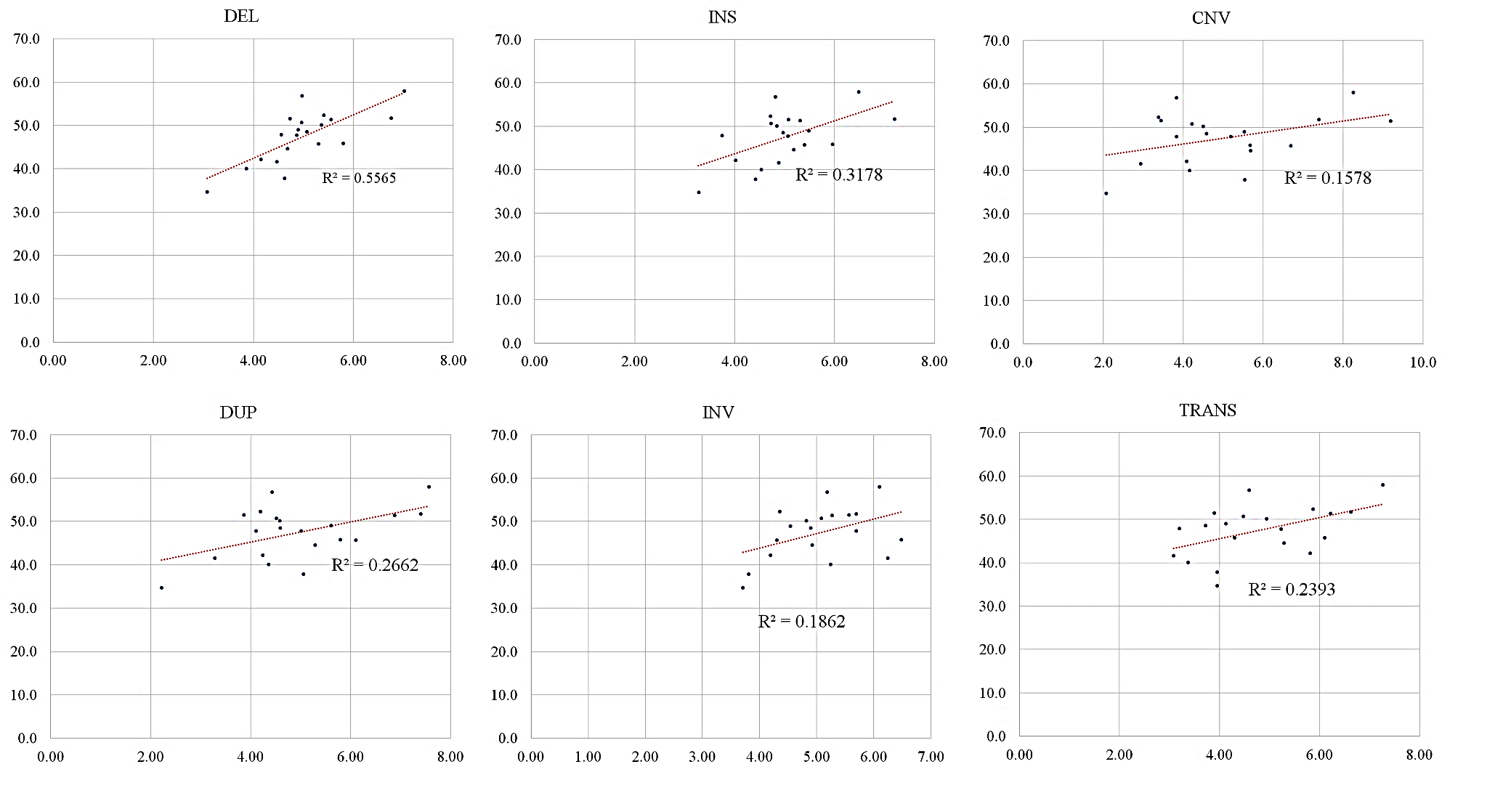


**Supplementary Figure 5.** Different cases used to identify structural variants that could directly impact the function of a gene. (1) the SV resides entirely within a gene, (2 and 3) a SV encompasses at least part of a gene or one of its breakpoints lies within a gene (4) the SV completely encompasses a gene.


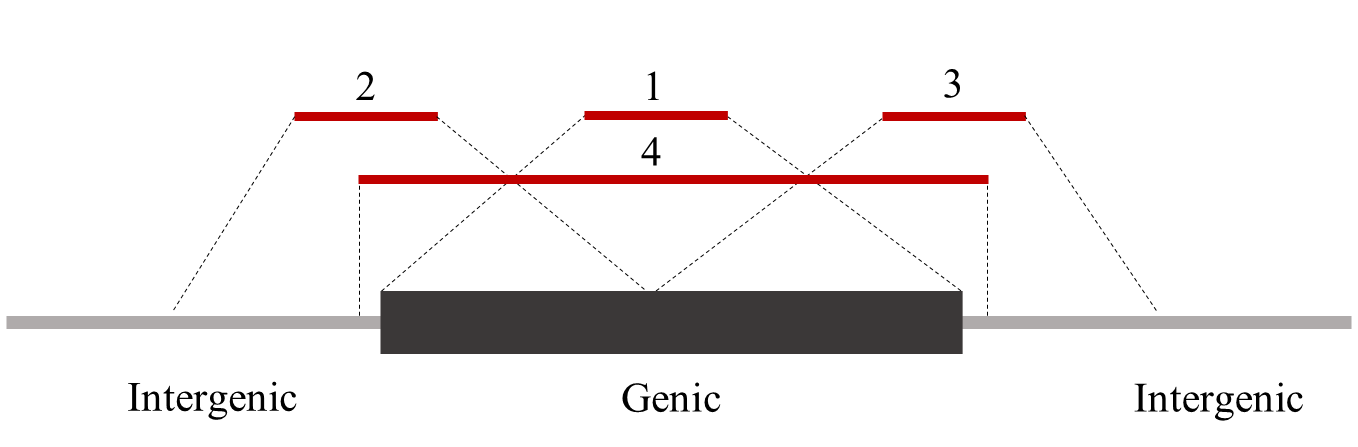


**Supplementary Figure 6.** Visualized example of PCR-based genotyping of 10 samples for *E4* gene. *E4* is the wild type form and *e4* resides an insertion. These results also confirmed the WGS SV genotypes dataset.


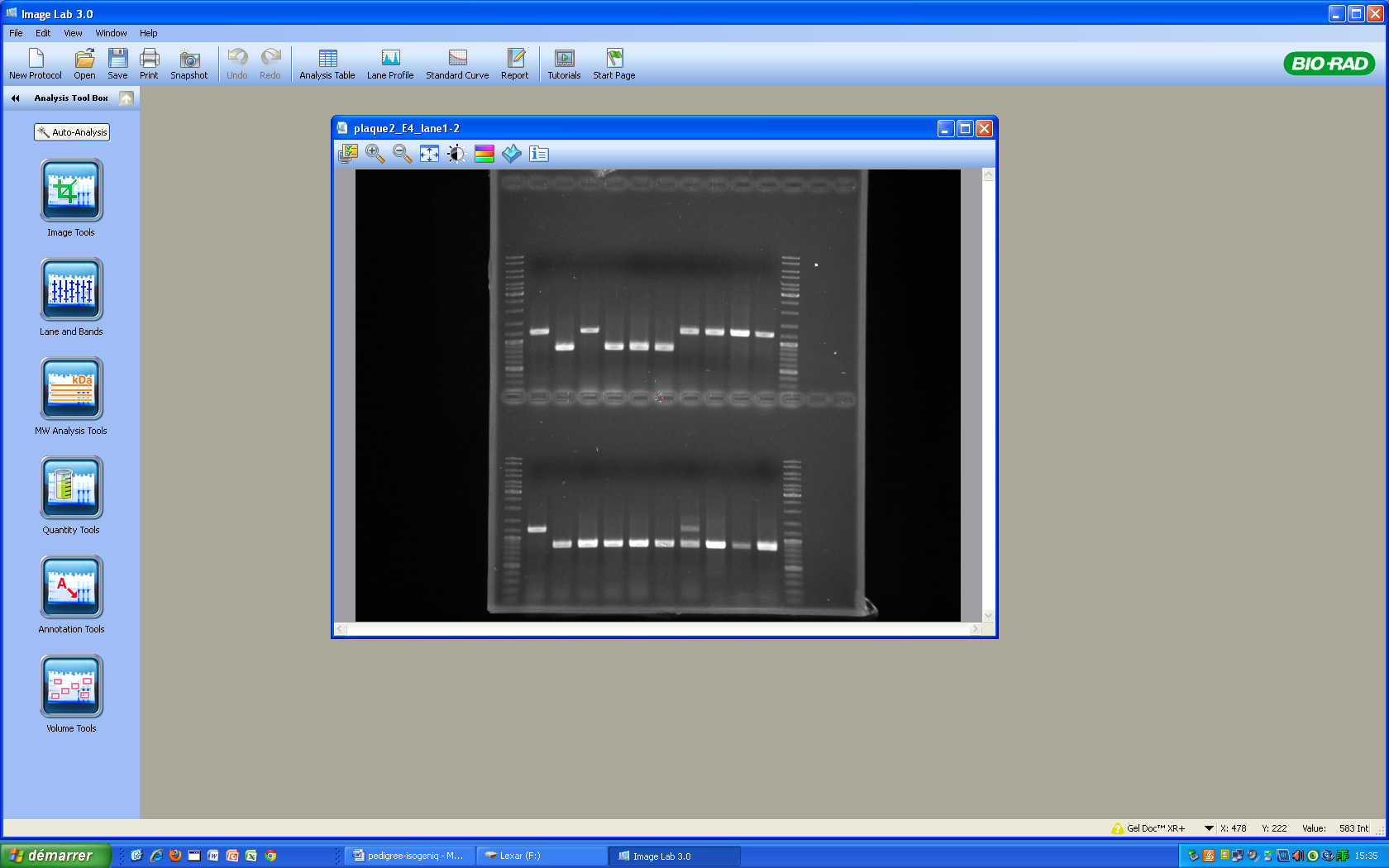
 **E4 e4**

**Supplementary Tables**

**Supplementary Table 1.** Information of sequenced short-season soybean accessions with name and number of trimmed reads (Phred score >32).

| **#** | **ID** | **Name** | **Year*** | **MG†** | **Number of trimmed reads** | **Read length** | **Depth of coverage** | **NCBI ID** |
| --- | --- | --- | --- | --- | --- | --- | --- | --- |
| **1** | CAD_1001 | OAC Bayfield | 1993 | 0 | 109,672,483 | 100 | 11x | SRX2403329 |
| **2** | CAD_1002 | OAC Prudence | 1999 | 0 | 73,089,815 | 100 | 8x | SRX2403301 |
| **3** | CAD_1003 | OAC Wallace | 2003 | 0 | 84,383,446 | 100 | 9x | SRX2403268 |
| **4** | CAD_1004 | AC Orford | 1999 | 0 | 101,157,223 | 100 | 10x | SRX2403327 |
| **5** | CAD_1005 | Gaillard | 1997 | 0 | 146,254,481 | 100 | 15x | SRX2403309 |
| **6** | CAD_1006 | OAC Prodigy | 2005 | I | 85,933,275 | 100 | 9x | SRX2403313 |
| **7** | CAD_1007 | AC Proteina | 1999 | 0 | 104,685,144 | 100 | 11x | SRX2403218 |
| **8** | CAD_1008 | Mandarin | 1934 | 0 | 99,895,528 | 100 | 10x | SRX2403240 |
| **9** | CAD_1009 | Evans | 1974 | 0 | 83,102,666 | 100 | 9x | SRX2403272 |
| **10** | CAD_1010 | Maple Presto | 1979 | 0 | 79,695,572 | 100 | 8x | SRX2403233 |
| **11** | CAD_1011 | OAC Ayton | 2006 | 0 | 81,394,689 | 100 | 8x | SRX2403255 |
| **12** | CAD_1012 | OAC Kent | 2000 | II | 99,135,548 | 100 | 10x | SRX2403326 |
| **13** | CAD_1013 | OAC Thames | 1993 | I | 72,834,364 | 100 | 7x | SRX2403315 |
| **14** | CAD_1014 | Dares | 1999 | 0 | 88,238,531 | 100 | 9x | SRX2403322 |
| **15** | CAD_1015 | OAC Lakeview | 2006 | 0 | 93,616,862 | 100 | 10x | SRX2403284 |
| **16** | CAD_1016 | OAC Morris | 2001 | 0 | 84,753,952 | 100 | 9x | SRX2403251 |
| **17** | CAD_1017 | Maple Glen | 1987 | 0 | 81,148,676 | 100 | 8x | SRX2403270 |
| **18** | CAD_1018 | Alta | 2001 | 0 | 89,081,938 | 100 | 9x | SRX2403304 |
| **19** | CAD_1019 | OAC Champion | 2008 | 0 | 82,264,388 | 100 | 8x | SRX2403271 |
| **20** | CAD_1020 | OT94-47 | BRL | 0 | 72,327,416 | 100 | 7x | SRX2403241 |
| **21** | CAD_1021 | OAC Erin | 1996 | 0 | 82,861,641 | 100 | 9x | SRX2403247 |
| **22** | CAD_1022 | OAC Pétrel/OAC 07-03C | 2012 | 0 | 88,990,425 | 100 | 9x | SRX2403239 |
| **23** | CAD_1023 | Majesta | 2003 | I | 82,752,204 | 100 | 8x | SRX2403234 |
| **24** | CAD_1025 | 12S01C-057 | BRL | I-II | 438,761,357 | 125 | 45x | SRX2403262 |
| **25** | CAD_1026 | 12S04C-043 | BRL | I-II | 60,411,245 | 125 | 8x | SRX2403242 |
| **26** | CAD_1027 | QS06099.38J | BRL | 0-I | 80,248,414 | 125 | 10x | SRX2403331 |
| **27** | CAD_1028 | QS06099.38Jb | BRL | 0-I | 81,861,222 | 125 | 11x | SRX2403265 |
| **28** | CAD_1029 | QS06009.25J | BRL | 0-I | 73,460,192 | 125 | 9x | SRX2403227 |
| **29** | CAD_1030 | QS06012.30J | BRL | 0-I | 164,774,241 | 125 | 21x | SRX2403224 |
| **30** | CAD_1031 | QS06017.35j | BRL | 0-I | 54,436,978 | 125 | 7x | SRX2403266 |
| **31** | CAD_1032 | 12S01C-074 | BRL | I-II | 34,204,143 | 125 | 4x | SRX2403305 |
| **32** | CAD_1033 | 12S01C-075 | BRL | I-II | 84,473,329 | 125 | 11x | SRX2403285 |
| **33** | CAD_1034 | QS06008.6J | BRL | 0-I | 52,446,525 | 125 | 7x | SRX2403324 |
| **34** | CAD_1035 | 12S01C-045 | BRL | I-II | 26,575,376 | 125 | 3x | SRX2403244 |
| **35** | CAD_1036 | 12S02C-024 | BRL | I-II | 72,536,650 | 125 | 9x | SRX2403248 |
| **36** | CAD_1037 | QS06094.7J | BRL | 0-I | 87,195,772 | 125 | 11x | SRX2403246 |
| **37** | CAD_1038 | 12S02C-026 | BRL | I-II | 23,194,296 | 125 | 3x | SRX2403319 |
| **38** | CAD_1039 | CERM13.5 | BRL | 0-I | 150,587,827 | 125 | 19x | SRX2403311 |
| **39** | CAD_1040 | X5302-1-52-3-2-B | BRL | 0 | 105,539,060 | 125 | 14x | SRX2403316 |
| **40** | CAD_1041 | X5346-1-3-4-4-B | BRL | 0 | 84,976,767 | 125 | 11x | SRX2403306 |
| **41** | CAD_1042 | QS06087.4J | BRL | 0-I | 56,271,105 | 125 | 7x | SRX2403332 |
| **42** | CAD_1043 | QS07001-6 | BRL | 0-I | 38,275,734 | 125 | 5x | SRX2403243 |
| **43** | CAD_1044 | X5377-1-43-4-2-B | BRL | 0 | 99,507,793 | 125 | 13x | SRX2403238 |
| **44** | CAD_1045 | X5367-1-96-4-2-B | BRL | 0 | 136,532,801 | 125 | 18x | SRX2403314 |
| **45** | CAD_1046 | QS4003.28B | BRL | 0 | 28,613,522 | 125 | 4x | SRX2403286 |
| **46** | CAD_1047 | OAC Sparta | 1993 | 0 | 149,809,944 | 125 | 19x | SRX2403269 |
| **47** | CAD_1048 | OAC Inwood | 1998 | I | 173,020,046 | 125 | 22x | SRX2403259 |
| **48** | CAD_1049 | OAC Oxford | 1999 | 0 | 127,434,932 | 125 | 16x | SRX2403236 |
| **49** | CAD_1050 | QS4004P.4J | BRL | 0 | 98,991,668 | 125 | 13x | SRX2403274 |
| **50** | CAD_1051 | Altesse | 1999 | 0 | 179,107,674 | 125 | 23x | SRX2403328 |
| **51** | CAD_1052 | OAC Embro | 1995 | I | 279,155,350 | 125 | 36x | SRX2403252 |
| **52** | CAD_1053 | OAC Ginty | 2009 | I | 213,037,560 | 125 | 27x | SRX2403258 |
| **53** | CAD_1054 | OAC Glencoe | 1995 | II | 76,659,274 | 125 | 10x | SRX2403221 |
| **54** | CAD_1055 | OAC Clinton | 1999 | 0 | 103,790,367 | 125 | 13x | SRX2403273 |
| **55** | CAD_1056 | OAC Drayton | 2010 | 0 | 134,506,966 | 125 | 17x | SRX2403225 |
| **56** | CAD_1057 | QS5055.43G | BRL | 0 | 129,482,183 | 125 | 17x | SRX2403254 |
| **57** | CAD_1058 | OAC Atwood | 1996 | 0 | 64,261,984 | 125 | 8x | SRX2403303 |
| **58** | CAD_1059 | OAC Huron | 2005 | I | 32,969,000 | 125 | 4x | SRX2403231 |
| **59** | CAD_1060 | OAC07-06C | BRL | 0 | 85,256,349 | 125 | 11x | SRX2403263 |
| **60** | CAD_1061 | QS4039P.15B(G) | BRL | 00-0 | 148,718,155 | 125 | 19x | SRX2403228 |
| **61** | CAD_1062 | OAC Salem | 1993 | 0 | 104,410,975 | 125 | 13x | SRX2403256 |
| **62** | CAD_1063 | OAC Bright | 2004 | 0 | 60,296,434 | 125 | 8x | SRX2403302 |
| **63** | CAD_1064 | OAC Carman | 2006 | 0 | 106,682,743 | 125 | 14x | SRX2403291 |
| **64** | CAD_1065 | OAC Madoc | 2010 | 0 | 103,830,072 | 125 | 13x | SRX2403253 |
| **65** | CAD_1066 | QS4067P.17j | BRL | 0-I | 190,764,155 | 125 | 24x | SRX2403232 |
| **66** | CAD_1067 | QS5085.8Bp | BRL | 0 | 81,908,340 | 125 | 11x | SRX2403229 |
| **67** | CAD_1068 | OAC Eclipse | 1989 | 0 | 131,113,766 | 125 | 17x | SRX2403249 |
| **68** | CAD_1069 | QS4042.6Bp | BRL | 0 | 186,241,009 | 125 | 24x | SRX2403219 |
| **69** | CAD_1070 | QS5091.50j | BRL | 0-I | 33,183,152 | 125 | 4x | SRX2403278 |
| **70** | CAD_1071 | OAC Gretna | 2006 | 0 | 113,259,243 | 125 | 15x | SRX2403293 |
| **71** | CAD_1072 | DH618(OAC06-18) | 2011 | 0 | 76,827,664 | 125 | 10x | SRX2403264 |
| **72** | CAD_1073 | Bloomfield | 2010 | 0 | 114,936,261 | 125 | 15x | SRX2403300 |
| **73** | CAD_1074 | OAC Stratford | 1999 | I | 191,957,047 | 125 | 25x | SRX2403275 |
| **74** | CAD_1075 | OT11-01 | BRL | 0 | 72,435,459 | 125 | 9x | SRX2403260 |
| **75** | CAD_1076 | SECAN09-42C | BRL | I | 174,934,164 | 125 | 22x | SRX2403276 |
| **76** | CAD_1077 | AC2001 | 1997 | 0 | 165,302,239 | 125 | 21x | SRX2403330 |
| **77** | CAD_1078 | Kamichis | 2003 | 0 | 71,364,991 | 125 | 9x | SRX2403299 |
| **78** | CAD_1079 | AC Proteus | 1993 | 0 | 129,473,344 | 125 | 17x | SRX2403222 |
| **79** | CAD_1080 | Acme | 1953 | 0 | 83,664,508 | 125 | 11x | SRX2403237 |
| **80** | CAD_1081 | Maple Amber | 1981 | 0 | 161,327,656 | 125 | 21x | SRX2403312 |
| **81** | CAD_1082 | QGC16T | 2007 | 0 | 83,783,463 | 125 | 11x | SRX2403290 |
| **82** | CAD_1083 | AC Bravor | 1990 | 0 | 117,420,669 | 125 | 15x | SRX2403230 |
| **83** | CAD_1084 | Morsoy | 1970 | 0 | 152,232,320 | 125 | 20x | SRX2403235 |
| **84** | CAD_1085 | Rodeo | 2004 | 0 | 42,194,259 | 125 | 5x | SRX2403307 |
| **85** | CAD_1086 | OT05-18 | BRL | 0 | 133,587,055 | 125 | 17x | SRX2403317 |
| **86** | CAD_1087 | Roland | 2005 | 0 | 124,753,190 | 125 | 16x | SRX2403292 |
| **87** | CAD_1088 | SECAN08-24C | 2012 | I | 135,866,218 | 125 | 17x | SRX2403283 |
| **88** | CAD_1089 | OAC09-35C | BRL | I | 43,152,099 | 125 | 6x | SRX2403267 |
| **89** | CAD_1090 | 9004 | 1996 | 0 | 47,484,470 | 125 | 6x | SRX2403296 |
| **90** | CAD_1091 | Heather | 2010 | 0 | 84,075,803 | 125 | 11x | SRX2403308 |
| **91** | CAD_1092 | Maple Isle | 1984 | 0 | 103,737,443 | 125 | 13x | SRX2403223 |
| **92** | CAD_1093 | 9063 | 1995 | 0 | 57,413,934 | 125 | 7x | SRX2403325 |
| **93** | CAD_1094 | OAC Eramosa | 1993 | 0 | 42,969,516 | 125 | 6x | SRX2403261 |
| **94** | CAD_1095 | Jari | 2010 | 0 | 34,578,831 | 125 | 4x | SRX2403245 |
| **95** | CAD_1096 | OT09-03 | BRL | 0 | 71,229,002 | 125 | 9x | SRX2403310 |
| **96** | CAD_1097 | Pagoda | 1939 | 0 | 110,673,635 | 125 | 14x | SRX2403318 |
| **97** | CAD_1098 | OT02-18 | BRL | 0 | 80,379,804 | 125 | 10x | SRX2403250 |
| **98** | CAD_1099 | Garry | 2016 | 0 | 71,322,061 | 125 | 9x | SRX2403220 |
| **99** | CAD_1100 | Dundas | 2004 | 0 | 46,664,364 | 125 | 6x | SRX2403323 |
| **100** | CAD_1101 | Maple Donovan | 1986 | 0 | 58,065,435 | 125 | 7x | SRX2403226 |
| **101** | CAD_1102 | Toki | 2008 | 0 | 54,066,561 | 125 | 7x | SRX2403287 |
| **102** | CAD_1103 | Katrina | 2005 | I | 53,617,720 | 125 | 7x | SRX2403257 |

*The year of introduction of the cultivar. BRL: breeding line (advanced line)

†MG: maturity group

**Supplementary Table 2.** Primers used for PCR-based SV validation.

| **#** | **Primer F/R** | **Primer Sequence** |
| --- | --- | --- |
| 1 | Forward | TTGGTCAAACGCATCCCAGAATTGC |
|  | Reverse1 | CCCATAGAGAGAGGTCGACAGCGTA |
|  | Reverse2 | ACAAGATGTGCCCAATACCCAACCG |
| 2 | Forward | ACGTATGTAAAGTTTGCAAGTCATAGC |
|  | Reverse1 | ACAAGATGTGCCCAATACCCAACCG |
|  | Reverse2 | TTCCCCATAGAGAGAGGTCGACAGC |
| 3 | Forward1 | ACAAAACAAGGCACTCCACATGAAT |
|  | Forward2 | AGCAATAGTTTATGGTGAAAGGGTTA |
|  | Reverse | GGCAGTCAGACCAATTGATAAGGT |
| 4 | Forward1 | AGCTCTTATTGAGGCAGACATGCAG |
|  | Forward2 | ATTACACTGCATATCACACGCAACG |
|  | Reverse | TAAGCTAAGGGATCACCATTCACCA |
| 5 | Forward1 | TGTATTTGGCTTTCCGTTATTAGTACA |
|  | Forward2 | TCGACAATGGTGCAAGAATCTGATA |
|  | Reverse | ATAGAATGGAACAACGTCGGGATCT |
| 6 | Forward1 | ACCTATTGGGCTAGGTTGGGCTAGT |
|  | Forward2 | TGTTGTTGCAGTACCCAGAGAAATG |
|  | Reverse | TATATACCGGGTCAAATCGGGCTAA |
| 7 | Forward1 | AAGACCCATTAGGAACAACTTTAATGA |
|  | Forward2 | ATTTCAAATGAAGGCCCAAAAGAGA |
|  | Reverse | CAGGTGAATCTTTCACAGGAATGCT |
| 8 | Forward1 | GCGAAAATGTTCAATTTTCAAAAGG |
|  | Forward2 | TGGCCTTTCATATCTCTGTTGGAAA |
|  | Reverse | CCGTATGAGGTGGAATTGAGAAACA |
| 9 | Forward1 | TCTCAAGAGGAAGAAGAAAATGAAAC |
|  | Forward2 | GAAGATGATGATGGGAACATTTGGA |
|  | Reverse | GAAACTTTGCTTTGCAATACATTCAT |
| 10 | Forward1 | ACCTATTGGGCTAGGTTGGGCTAGT |
|  | Forward2 | ACCACCGTTTTTGCCTTATCATTTC |
|  | Reverse | TCAGTCCGACCCGACTATTTTTACA |
| 11 | Forward1 | TGAAATGGGTTTATGGAATGGAGGT |
|  | Forward2 | TTCTCGGGACGAGATCATACACCTA |
|  | Reverse | AAATGATATTGCTGCTGAAGGCTAA |
| 12 | Forward1 | TTACCCTCACCAATTCCCAGTACCT |
|  | Forward2 | CAGTTATTACGGTACAAGGCCGTCA |
|  | Reverse | TCGATCTCCCTTGCTTTTTCTATTT |
| 13 | Forward1 | CATGAAATTCAGCAATCAGTCAAAA |
|  | Forward2 | TGGACTGTCTTTATACCTCCCACGA |
|  | Reverse | CATGAAATTCAGCAATCAGTCAAAA |
| 14 | Forward | TGTTTGAGCGCCTCTATTTGGACTA |
|  | Reverse1 | TGAACTTTCAACGACACTCGATTCA |
|  | Reverse2 | GAAAAGCATAAAATTTTCGTCGAGCTA |
| 15 | Forward | CGAGACAACCTGATTTACACCCTCT |
|  | Reverse1 | TCAGTCCGACCCGACTATTTTTACA |
|  | Reverse2 | TTGACGCTGTTGGACTCTCTAGTCA |
| 16 | Forward | GCGTGCTTCCGAGGCAAAAATACCT |
|  | Reverse1 | GCCGGAACATGAGAAAATTACTAATGG |
|  | Reverse2 | GCCTTATTTGAAAGATGGAAGACCCGT |
| 17 | Forward | ACCAAACCAACAACTATGACACTGGA |
|  | Reverse1 | ACAGCGTTGCGGACTATAGCAGAAG |
|  | Reverse2 | CTCCGGCCCTCCAATGAACATTAGT |
| 18 | Forward | ATCTTTGCTTGATCTTTTGCGTCAA |
|  | Reverse1 | AACGACACTCGATTCAACAACAGTTGC |
|  | Reverse2 | AGTTGCACCATATCATTTGCTAGCGA |
| 19 | Forward | GTCTGTCACCTCCTTGCAATGCGTC |
|  | Reverse1 | TATGGCTTCCATGGTGGCTTGGTTC |
|  | Reverse2 | TTGCTGGCTCAAAGAAGGCACTGTT |
| 20 | Forward | AGCTGGTGGAACAAGACTCTGAAGG |
|  | Reverse1 | AGATTGACTGTTACTCACGAGGCTGT |
|  | Reverse2 | ATAACTAGTTGGTAACCCGTGCGTA |
| 21 | Forward | GAAGTTCTCCAAAATTTACAGGGAGA |
|  | Reverse | TGTGAGTCAAGGAAATCCCAACATT |
| 22 | Forward | GAAGTTACAAAACTAAGCCGCATTG |
|  | Reverse | CACAGGTCTCACAAATACGTGGAGT |
| 23 | Forward | CTCGGATCTTGACAGCATCA |
|  | Reverse | GTCCTATACAATTCTTTACGACG |
| 24 | Forward | TGCTATATGAAAGCATTCGGGGTAT |
|  | Reverse | TGTTTTGTGGGTGGGAAAGGTTAGGT |
| 25 | Forward | AAGGTTACTTGTGTCATTGTGTGGT |
|  | Reverse | TGTGTCTTTAGTATGAGTGTCACCAA |
| 26 | Forward | TGGGTTATTCTTCCTGCACCATTCA |
|  | Reverse | ACGCTAAATGAAAGGTATGTTGAATGT |
| 27 | Forward | GGATCCGGAGATGCACCTCTATTCAT |
|  | Reverse1 | GCACGCATTTTTGTGTAGCAACAAGA |
|  | Reverse2 | ACGAGTTCCTGTCCTGGTTTACCAT |
| 28 | Forward | TCAAGCAGCTGGAGTTTGGTTTCCA |
|  | Reverse1 | ATCTAAACCCATGGTTGTTGCCGTG |
|  | Reverse2 | ACACTTCGACAAGAGTTTCCGAAAC |
| 29 | Forward | TGAATCTCGAACACGTTTCATCGAA |
|  | Reverse1 | TCAAAAGTCAAGCCAAGTATAGATGTGT |
|  | Reverse2 | AGGAAGAAAGAAAACGAAAACTTATGAGT |
| 30 | Forward | TGCCATTAAAATTCTTTAACTAACGGAGT |
|  | Reverse | TCAAGACAGCCTCTGTGTTTGTAGCT |
| 31 | Forward | AATGGTCGCCTATTACACGGCTCAC |
|  | Reverse1 | AGTGGAAGGGTGAGTCAGAGACTGA |
|  | Reverse2 | AGCACCTACCCACGGGAAGATAGAA |
| 32 | Forward | CCTCTCCCTCCACTCATCTTCTCCT |
|  | Reverse1 | AGGATGTGTGAAAGAAGGCACAGGC |
|  | Reverse2 | AGGATGTAGTAGCCAGGTGTGTGGT |
| 33 | Forward | TTCCATGGAGGCATCAACTTTGGCC |
|  | Reverse1 | ACTTGCACAGCTACGAGTTGACGTT |
|  | Reverse2 | GTGAGACCCGTACCTGCTTTGACTC |
| 34 | Forward | GGTGTAATTGAGGGGTTTAGTGATGCA |
|  | Reverse | AAGGAACCTTTCGAGCTTGAATCTT |
| 35 | Forward | GTGAACTCAACCGCAATTGGTGTCA |
|  | Reverse1 | TGGGGTTTACCATGTTGACAGACCA |
|  | Reverse2 | ACCAAAGGAACGCCTAAACCATCGT |
| 36 | Forward | TGACTCTGGTGTGGATAAGGGTGAT |
|  | Reverse1 | ACCATAGGAAGCCATGGATAAGAGC |
|  | Reverse2 | TTATTCTGGAGGGAGAAGATGGTGA |
| 37 | Forward | TGTTTGGATACCTGTATCTGAAAATTGA |
|  | Reverse1 | TGCTCTTGCATCGTATACCCAAGTT |
|  | Reverse2 | ATTATTGAGGTGCTCCGAAAGGTTC |
| 38 | Forward | ATGCACTAACCATGTTGCAGGAAGT |
|  | Reverse1 | ACCATGTTCTACCATGAAGTGACATT |
|  | Reverse2 | AAGGAGTCGGAGGGAAGTACGGATA |
| 39 | Forward | AGACGTAGTGCTAGGGCTAT |
|  | Reverse | GCTCATCCCTTCGAATTCAG |
| 40 | Forward | AGACGTAGTGCTAGGGCTAT |
|  | Reverse | GCTCATCCCTTCGAATTCAG |

**Supplementary Table 3.** List of genes containing variants predicted to have a high impact on gene function.

| **Gene ID** | **Length** | **Database** | **Annotation** | **Functional homolog** |
| --- | --- | --- | --- | --- |
| **Glyma.01G003500** | 2121 | PTHR | N Myc Down regulated Like 3 Protein | ✓ |
| **Glyma.01G012200** | 3260 | PTHR | CGI 141_Related | ✓ |
| **Glyma.01G018100** | 899 |  |  | _ |
| **Glyma.01G033800** | 3685 | Pfam | EamA Like Transporter Family (Eama) | ✓ |
| **Glyma.01G073700** | 1803 | KOG | DNA Helicase Pif1 | ✓ |
| **Glyma.01G108600** | 1466 | AT | Ditrans Polycis Polyprenyl Diphosphate Synthase (2E6E) Farnesyl Diphosphate Specific | ✓ |
| **Glyma.01G215400** | 973 | AT | dTDP 4 dehydrorhamnose reductase | ✓ |
| **Glyma.01G230500** | 2303 | Pfam | PPR Repeat (PPR) | ✓ |
| **Glyma.01G241200** | 1851 | PTHR | Protein_Related | ✓ |
| **Glyma.02G022800** | 6787 | PTHR | Genomic DNA Chromosome 3 P1 Clone:Msd24 | ✓ |
| **Glyma.02G033600** | 7781 | PTHR | Potassium Transporter 6 | ✓ |
| **Glyma.02G065000** | 4223 |  |  | _ |
| **Glyma.02G123300** | 6928 | PTHR | Geranylgeranyl Transferase Type 2 Subunit Alpha | × |
| **Glyma.02G182700** | 2186 | Pfam | PPR Repeat (PPR) | ✓ |
| **Glyma.02G215000** | 4664 | PTHR | Vernalization Insensitive Protein 3 | ✓ |
| **Glyma.02G219300** | 8957 | PTHR | Chaperone Activity Of Bc1 Complex CABC1 _Related | ✓ |
| **Glyma.02G292200** | 13704 | PTHR | Muts Protein Homolog 5 | ✓ |
| **Glyma.02G294600** | 4976 | PTHR | Grpe Protein Homolog Mitochondrial | ✓ |
| **Glyma.02G295000** | 5877 | PTHR | Phosphatidate Cytidylyltransferase | ✓ |
| **Glyma.02G311400** | 536 | PTHR | Calcium Binding Ef Hand Containing Protein | × |
| **Glyma.03G031500** | 3264 | PTHR | Spindle And Kinetochore Associated Protein 1 | ✓ |
| **Glyma.03G043900** | 4710 | PTHR | Leucine Rich Repeat Containing Protein | ✓ |
| **Glyma.03G059000** | 3282 | KOG | DNA Helicase Pif1 | ✓ |
| **Glyma.03G065600** | 4364 | PTHR | Potassium Channel Tetramerization Domain Containing | ✓ |
| **Glyma.03G066700** | 3359 | PTHR | Aldo Keto Reductase Family 4 Member C10 | ✓ |
| **Glyma.03G069500** | 2850 | Pfam | MULE Transposase Domain (Mule) | ✓ |
| **Glyma.03G071100** | 3290 | AT | Phosphatidylserine Decarboxylase | × |
| **Glyma.03G101200** | 9826 | PTHR | Protein Eceriferum 1 | ✓ |
| **Glyma.03G122700** | 578 | PTHR | CBL Interacting Serine | ✓ |
| **Glyma.03G124700** | 4317 | PTHR | Mediator Of Rna Polymerase Ii Transcription Subunit 26A_Related | ✓ |
| **Glyma.03G146100** | 1272 | KOG | Uncharacterized Conserved Protein | ✓ |
| **Glyma.03G169400** | 4536 | PTHR | Sugar Kinase | ✓ |
| **Glyma.03G177300** | 3464 | PTHR | Protein Reveille 4_Related | ✓ |
| **Glyma.03G218900** | 927 | Pfam | Domain Of Unknown Function (Duf4228) (Duf4228) | ✓ |
| **Glyma.03G241800** | 595 | KOG | Histone 2A | ✓ |
| **Glyma.03G257400** | 445 |  |  | _ |
| **Glyma.04G013500** | 1721 | Pfam | BURP domain (BURP) | ✓ |
| **Glyma.04G034300** | 828 | PTHR | Pathogenesis_Relatedprotein 5_Related | ✓ |
| **Glyma.04G062800** | 1018 | Pfam | Plant Protein Of Unknown Function (Duf868) (Duf868) | ✓ |
| **Glyma.04G075700** | 1683 | PTHR | F Box Protein AFR | ✓ |
| **Glyma.04G086500** | 7485 | AT | Tryptophan tRNA ligase | ✓ |
| **Glyma.04G112400** | 1487 |  |  | _ |
| **Glyma.04G207200** | 5222 | Pfam | Polyketide Cyclase | ✓ |
| **Glyma.04G227000** | 4239 | PTHR | Heavy Metal Transport | ✓ |
| **Glyma.04G242900** | 5223 | AT | Non Specific Serine | ✓ |
| **Glyma.05G077100** | 4652 | PTHR | Chloride Channel Protein CLC_A_Related | ✓ |
| **Glyma.05G109400** | 16694 | PTHR | Lysosomal Pro X Carboxypeptidase | ✓ |
| **Glyma.05G126300** | 1226 | Pfam | Plant Protein Of Unknown Function (Duf247) | ✓ |
| **Glyma.05G137700** | 1853 |  |  | _ |
| **Glyma.05G185900** | 2088 |  |  | _ |
| **Glyma.05G202400** | 1207 | KOG | Mitochondrial Import Inner Membrane Translocase Subunit Tim8 | ✓ |
| **Glyma.05G203600** | 1314 | PTHR | HVA22 Like Proteins | ✓ |
| **Glyma.05G206700** | 1039 | Pfam | Haloacid Dehalogenase Like Hydrolase (Hydrolase) | × |
| **Glyma.05G206800** | 302 |  |  | _ |
| **Glyma.05G207100** | 2410 | PTHR | Glutathione Peroxidase | ✓ |
| **Glyma.05G213500** | 1030 |  |  | _ |
| **Glyma.05G230800** | 1189 | PTHR | Genomic DNA Chromosome 3 P1 Clone: Mob24 | × |
| **Glyma.05G233800** | 1003 | PTHR | AAA Type Atpase Family Protein_Related | × |
| **Glyma.05G236100** | 2897 | PTHR | NADH Dehydrogenase [Ubiquinone] Iron Sulfur Protein 8 | ✓ |
| **Glyma.06G036000** | 1482 | PTHR | Leucine Rich Repeat Extensin Like Protein 3_Related | ✓ |
| **Glyma.06G072800** | 1216 | PTHR | O Glycosyl Hydrolases Family 17 Protein | × |
| **Glyma.06G081400** | 4369 | PTHR | Ribosomal S Subunit | ✓ |
| **Glyma.06G203700** | 3719 | PTHR | EMB | ✓ |
| **Glyma.06G204900** | 1064 | PTHR | Mitochondrial Transcription Termination Factor Family Protein | × |
| **Glyma.06G244400** | 2040 | Pfam | Replication Factor A C Terminal Domain (Rep_Fac A_C) | ✓ |
| **Glyma.06G273900** | 6756 | AT | Actinidain | ✓ |
| **Glyma.06G274400** | 2008 |  |  | _ |
| **Glyma.06G274800** | 951 |  |  | _ |
| **Glyma.06G292400** | 4660 | PTHR | Mannan Endo 1 4 Beta Mannosidase 2_Related | ✓ |
| **Glyma.06G299600** | 3445 | PTHR | Equilibrative Nucleotide Transporter 2 | ✓ |
| **Glyma.06G300000** | 1684 | PTHR | MYB Like DNA Binding Protein Myb | ✓ |
| **Glyma.07G014700** | 950 | AT | Acid Phosphatase | × |
| **Glyma.07G015700** | 6222 | PTHR | Malic Enzyme_Related | ✓ |
| **Glyma.07G033600** | 1449 | PTHR | Oxidoreductase 2Og Fe Ii Oxygenase Family Protein | ✓ |
| **Glyma.07G068500** | 3295 | Pfam | PPR Repeat (PPR) | ✓ |
| **Glyma.07G105800** | 1295 | Pfam | PPR Repeat (PPR) | ✓ |
| **Glyma.07G107200** | 1024 | PTHR | Zinc Finger Homeodomain Protein 14 | ✓ |
| **Glyma.07G124600** | 2036 | PTHR | Mate Efflux Family Protein | ✓ |
| **Glyma.07G127000** | 1344 | PTHR | SNF7 Related | ✓ |
| **Glyma.07G130100** | 4161 | Pfam | Cation Transport Protein (Trkh) | ✓ |
| **Glyma.07G142200** | 228 |  |  | _ |
| **Glyma.07G143300** | 691 | Pfam | Protein Of Unknown Function (Duf674) (Duf674) | × |
| **Glyma.07G164600** | 4422 | Pfam | Membrane Transport Protein (Mem_Trans) | ✓ |
| **Glyma.07G205700** | 1686 | PTHR | Armadillo | × |
| **Glyma.07G211200** | 1606 | PTHR | Carboxylesterase 12_Related | ✓ |
| **Glyma.08G030300** | 2525 | PTHR | Regulator Of Vps4 Activity Protein_Related | × |
| **Glyma.08G038000** | 1120 |  |  | _ |
| **Glyma.08G053900** | 1640 | Pfam | PPR Repeat (PPR) | ✓ |
| **Glyma.08G061400** | 1174 | Pfam | Protein Of Unknown Function Duf538 (Duf538) | × |
| **Glyma.08G098100** | 4179 | Pfam | PPR Repeat (PPR) | ✓ |
| **Glyma.08G176300** | 1749 | PTHR | Cis Epoxycarotenoid Dioxygenase NCED3 Chloroplastic_Related | ✓ |
| **Glyma.08G255500** | 2817 | Pfam | PPR Repeat (PPR) | ✓ |
| **Glyma.08G322400** | 4444 | PTHR | Protein C05C9.1 | ✓ |
| **Glyma.08G323800** | 7324 | Pfam | Cytosine Specific DNA Methyltransferase Replication Foci Domain (Dnmt1 Rfd) | × |
| **Glyma.08G351500** | 846 | PTHR | EMB | ✓ |
| **Glyma.09G048400** | 1809 | PTHR | Peroxidase 7 | ✓ |
| **Glyma.09G067200** | 4606 | Pfam | LysM domain (LysM) | ✓ |
| **Glyma.09G081900** | 679 | PTHR | Protein Tom Three Homolog 1_Related | ✓ |
| **Glyma.09G122100** | 1089 | PTHR | Eukaryotic Translation Initiation Factor 2C | × |
| **Glyma.09G131200** | 643 | PTHR | Copper Transport Family Protein_Related | ✓ |
| **Glyma.09G148600** | 2790 | PTHR | Peroxidase 66 | ✓ |
| **Glyma.09G173800** | 2507 | PTHR | Inactive Receptor Kinase Rlk902_Related | ✓ |
| **Glyma.09G188700** | 6039 | PTHR | Sulfate Transporter 3.5_Related | ✓ |
| **Glyma.09G195500** | 1640 | PTHR | Pentatricopeptide Repeat Containing Protein | × |
| **Glyma.09G241700** | 1014 | PTHR | Aaa Type Atpase Family Protein_Related | ✓ |
| **Glyma.09G246800** | 3483 | PTHR | Circadian Protein Clock | ✓ |
| **Glyma.09G246900** | 384 |  |  | _ |
| **Glyma.10G037000** | 2920 | PTHR | Myb Like DNA Binding Protein Myb | ✓ |
| **Glyma.10G098500** | 911 | PTHR | Nadh Ubiquinone Oxidoreductase Chain 4 | ✓ |
| **Glyma.10G165000** | 21490 | PTHR | Transcriptional Regulator Atrx Homolog | ✓ |
| **Glyma.10G188500** | 4491 | PTHR | Ethylene Response Sensor 2_Related | ✓ |
| **Glyma.10G217000** | 5419 | PTHR | Calcium Dependent Protein Kinase 11_Related | ✓ |
| **Glyma.10g221500** | 21387 | PTHR | Protein Gigantea | × |
| **Glyma.10G254900** | 674 | PTHR | Agamous Like Mads Box Protein Agl62 | ✓ |
| **Glyma.10G261000** | 1591 |  |  | _ |
| **Glyma.10G265500** | 1894 | Pfam | PPR Repeat (PPR) | ✓ |
| **Glyma.11G029300** | 1938 |  |  | _ |
| **Glyma.11G095500** | 6635 | Pfam | Adaptin C Terminal Domain (Alpha_Adaptinc21 (Cnd1) | ✓ |
| **Glyma.11G125600** | 4774 | Pfam | Programmed cell death protein 7 (PDCD7) | ✓ |
| **Glyma.11G227500** | 847 | PTHR | Calcium Dependent Lipid Binding Domain Protein | ✓ |
| **Glyma.12G091100** | 1006 | PTHR | Pollen Ole E 1 Allergen And Extensin Family Protein | ✓ |
| **Glyma.12G137900** | 9445 | Pfam | Leucine Rich Repeat (LRR_3) | ✓ |
| **Glyma.12G138600** | 4829 | AT | Germacradienol Synthase | ✓ |
| **Glyma.12G140900** | 4279 | Pfam | Pif1 Like Helicase (Pif1) | ✓ |
| **Glyma.12G141500** | 1129 | PTHR | Flavodoxin Like Quinone Reductase 1 | ✓ |
| **Glyma.12G141600** | 3581 | PTHR | ALDO | × |
| **Glyma.12G226100** | 3020 | PTHR | Protein Phosphatase 2C 39_Related | ✓ |
| **Glyma.13G050500** | 8857 | Pfam | Leucine Rich Repeat N Terminal Domain (LRRnt_2) | ✓ |
| **Glyma.13G069600** | 6567 | PTHR | Senescence Associated Carboxylesterase 101 | ✓ |
| **Glyma.13G070400** | 5296 | Pfam | SecE | ✓ |
| **Glyma.13G070500** | 5898 | PTHR | Transcription Elongation Factor B Polypeptide 3 | ✓ |
| **Glyma.13G125200** | 1468 | KOG | Ras SuPPRessor Protein (Contains Leucine Rich Repeats) | ✓ |
| **Glyma.13G131500** | 978 |  |  | _ |
| **Glyma.13G166900** | 3075 | Pfam | S Locus Glycoprotein Domain (S_Locus_Glycop | ✓ |
| **Glyma.13G189800** | 993 | PTHR | Member Of 'Gdxg' Family Of Lipolytic Enzymes | ✓ |
| **Glyma.13G199400** | 1304 | PTHR | Aspartic Proteinase Cdr1_Related | ✓ |
| **Glyma.13G248700** | 3275 | PTHR | Atp Binding | ✓ |
| **Glyma.13G261000** | 15905 | PTHR | Callose Synthase 8_Related | ✓ |
| **Glyma.13G289200** | 1705 | PTHR | Glucosyl | ✓ |
| **Glyma.14G009600** | 7354 | PTHR | Cytosolic Enolase 3 | ✓ |
| **Glyma.14G012900** | 6703 | PTHR | X Box Transcription Factor_Related | ✓ |
| **Glyma.14G013500** | 4720 | AT | Adp Ribose Diphosphatas | ✓ |
| **Glyma.14G016700** | 3268 | Pfam | C2 Domain (C2) | ✓ |
| **Glyma.14G018500** | 3083 | PTHR | Protein Regulator Of Cytokinesis 1 Prc1_Related | ✓ |
| **Glyma.14G031600** | 8426 | PTHR | Nuclear Deih Boxhelicase_Related | ✓ |
| **Glyma.14G042400** | 2363 | Pfam | Leucine Rich Repeat (LRR_1) | ✓ |
| **Glyma.14G045200** | 2987 | PTHR | Heat Shock Family Protein | ✓ |
| **Glyma.14G048300** | 6677 | Pfam | Protein Kinase Domain (Pkinase) | ✓ |
| **Glyma.14G051700** | 738 |  |  | _ |
| **Glyma.14G053400** | 2689 | Pfam | Weak Chloroplast Movement Under Blue Light (Wembl) | ✓ |
| **Glyma.14G059600** | 3112 |  |  | _ |
| **Glyma.14G154500** | 9612 | PTHR | Protein Rbd 1 | ✓ |
| **Glyma.14G157800** | 4646 | Pfam | Protein Kinase Domain (Pkinase) | ✓ |
| **Glyma.14G166500** | 4268 | PTHR | Auxin Response Factor 17 | ✓ |
| **Glyma.15G038000** | 2352 | PTHR | Protein Kinase Apk1A Chloroplastic_Related | ✓ |
| **Glyma.15G076300** | 9036 | PTHR | Serine Protease_Related | ✓ |
| **Glyma.15G083300** | 4088 | PTHR | Splicing Factor 3B Subunit 1_Related | ✓ |
| **Glyma.15G109200** | 438 |  |  | _ |
| **Glyma.15G120600** | 1463 | PTHR | Wall Associated Receptor Kinase Like 1_Related | ✓ |
| **Glyma.15G124300** | 742 |  |  | _ |
| **Glyma.15G127600** | 8496 | PTHR | Tubulin Specific Chaperone E | ✓ |
| **Glyma.15G138100** | 1369 | Pfam | Ferritin Like Domain (Ferritin_2) | ✓ |
| **Glyma.15G158000** | 1876 |  |  | _ |
| **Glyma.15G163800** | 21218 | PTHR | Orf Protein_Related | ✓ |
| **Glyma.15G167200** | 5456 | Pfam | Pif1 Like Helicase (Pif1) | ✓ |
| **Glyma.15G169400** | 721 | AT | Nonspecific Serine | ✓ |
| **Glyma.15G170000** | 13288 | PTHR | Protein Kinase Family Protein | ✓ |
| **Glyma.15G171400** | 12031 | Pfam | DNA Directed Rna Polymerase Iii Subunit Rpc31 (Rna_Pol_3_Rpc31) | ✓ |
| **Glyma.15G171600** | 965 | PTHR | Expressed Protein_Related | ✓ |
| **Glyma.15G195300** | 625 | Pfam | Gh3 Auxin Responsive Promoter (GH3) | × |
| **Glyma.15G197900** | 509 | PTHR | Ethylene Responsive Transcription Factor ERF003 | ✓ |
| **Glyma.15G215200** | 413 |  |  | _ |
| **Glyma.15G215400** | 6588 | PTHR | Phospholipid Transporting Atpase 1 | ✓ |
| **Glyma.15G219600** | 4813 | PTHR | Phosducin_Related | ✓ |
| **Glyma.15G223600** | 15199 | AT | Cycloartenol Synthase | × |
| **Glyma.15G229000** | 1159 | PTHR | Duplicated Sant DNA Binding Domain Containing Protein | ✓ |
| **Glyma.15G232300** | 1559 | PTHR | Ribosome Recycling Factor | ✓ |
| **Glyma.15G233600** | 1236 | PTHR | E3 Ubiquitin Protein Ligase Keg | × |
| **Glyma.15G247100** | 1520 | PTHR | Leucine Rich Repeat Containing Protein | × |
| **Glyma.15G274400** | 3969 |  |  | _ |
| **Glyma.16G017300** | 5617 | PTHR | Protein Kinase Family Protein | ✓ |
| **Glyma.16G018400** | 12773 | PTHR | Ring Zinc Finger Containing Protein | ✓ |
| **Glyma.16G019100** | 369 | PTHR | Proprotein Convertase Subtilisin | × |
| **Glyma.16G019200** | 3662 | AT | Aspergillus Nuclease S(1) | ✓ |
| **Glyma.16G024500** | 3245 | PTHR | Cytochrome B561 | ✓ |
| **Glyma.16G050000** | 12828 | PTHR | Mitochondrial Phosphate Carrier Protein 3 Mitochondrial | ✓ |
| **Glyma.16G066200** | 3070 | PTHR | Succinate Dehydrogenase Iron Sulfur Protein | ✓ |
| **Glyma.16G075200** | 8814 | Pfam | Bacterial Extracellular Solute Binding Protein (Sbp_Bac_8) | ✓ |
| **Glyma.16G085200** | 3851 | PTHR | Leucine Rich Repeat Containing Protein | ✓ |
| **Glyma.16G100200** | 2475 | PTHR | Histone Lysine N Methyltransferase H3 Lysine 9 Specific Suvh7_Related | ✓ |
| **Glyma.16G121600** | 946 |  |  | _ |
| **Glyma.16G134200** | 1959 |  |  | _ |
| **Glyma.16G135800** | 1508 | PTHR | Adenylate Kinase 1 Mitochondrial_Related | × |
| **Glyma.16G172500** | 7711 | PTHR | Abc Transporter C Family Member 10 | ✓ |
| **Glyma.16G208600** | 3397 | KOG | Translation Initiation Factor 6 (Eif 6) | ✓ |
| **Glyma.16G209200** | 745 |  |  | _ |
| **Glyma.16G216600** | 1623 | Pfam | Transmembrane Protein 18 (Tmem18) | ✓ |
| **Glyma.16G216900** | 3034 | PTHR | Nucleotide Diphospho Sugar Transferase Domain Containing Protein_Related | ✓ |
| **Glyma.16G217200** | 5611 | PTHR | Sucrose Synthase 5 | ✓ |
| **Glyma.16G221000** | 2264 |  |  | _ |
| **Glyma.17G019600** | 1436 | PTHR | UDP Glucosyl Transferase 73B2_Related | ✓ |
| **Glyma.17G124500** | 3134 | PTHR | Translation Factor | ✓ |
| **Glyma.17G152300** | 1106 | Pfam | Purine Nucleobase Transmembrane Transport (Punut) | ✓ |
| **Glyma.17G216400** | 5610 | Pfam | Pif1 Like Helicase (Pif1) | ✓ |
| **Glyma.18G051500** | 818 | PTHR | Shn Shine DNA Binding | ✓ |
| **Glyma.18G096800** | 14863 | PTHR | Nucleolar Preribosomal Associated Protein 1 | ✓ |
| **Glyma.18G123400** | 4934 | PTHR | Sucrose Transport Protein Suc3 | ✓ |
| **Glyma.18G131500** | 6846 | PTHR | Zinc Metalloprotease Ybr074W_Related | × |
| **Glyma.18G134000** | 970 | PTHR | Cytochrome P450 89A2_Related | ✓ |
| **Glyma.18G134400** | 6034 | AT | Inositol Hexakisphosphate Kinase | × |
| **Glyma.18G142000** | 2433 | Pfam | Protein Of Unknown Function (Duf 659) (Duf659) | ✓ |
| **Glyma.18G146400** | 1670 | PTHR | Finger And Bah Motif Containing Putative Transcription Factor_Related | × |
| **Glyma.18G148300** | 1340 | AT | 3 Methyl 2 Oxobutanoate Dehydrogenase (2 Methylpropanoyl Transfer | × |
| **Glyma.18G152700** | 435 | KOG | Ubiquitin | ✓ |
| **Glyma.18G175900** | 1314 | PTHR | Bed Finger_Related | ✓ |
| **Glyma.18G179400** | 3046 | Pfam | FAR1 DNA Binding Domain (Far1) | ✓ |
| **Glyma.18G179700** | 375 | AT | Mitochondrial Processing Peptidase | × |
| **Glyma.18G184400** | 5900 | PTHR | ATP Dependent Protease Cereblon | × |
| **Glyma.18G185700** | 393 |  |  | _ |
| **Glyma.18G222900** | 2025 | AT | Geraniol 8 Hydroxylase | ✓ |
| **Glyma.18G237500** | 351 |  |  | _ |
| **Glyma.18G270200** | 5966 |  |  | _ |
| **Glyma.18G275600** | 4423 | PTHR | Phosphatidylinositol 3 Kinase_Relatedprotein Kinase | ✓ |
| **Glyma.19G070700** | 4412 | PTHR | Calcium Dependent Lipid Binding Domain Containing Protein | ✓ |
| **Glyma.19G074500** | 1688 | PTHR | Surfeit Locus Protein 6 | ✓ |
| **Glyma.19G080500** | 2162 | PTHR | Pentatricopeptide Repeat Repeat Containing Protein | ✓ |
| **Glyma.19G133700** | 3644 | PTHR | Phosphatidylinositol N Acetylglucosaminyltransferase Subunit Q | ✓ |
| **Glyma.19G133900** | 3496 | AT | Nitric Oxide Synthase (Nadph) | ✓ |
| **Glyma.19G164000** | 520 | PTHR | Ethylene Responsive Transcription Factor Erf098 | ✓ |
| **Glyma.19G179300** | 5208 | PTHR | ATP Dependent Rna Helicase Ddx52_Related | ✓ |
| **Glyma.19G220600** | 1905 | PTHR | Pectinesterase | ✓ |
| **Glyma.19G246300** | 3439 | Pfam | MuDR family transposase (DBD_Tnp_Mut) | ✓ |
| **Glyma.19G256100** | 1937 | PTHR | Ubiquitin | ✓ |
| **Glyma.20G050400** | 626 | PTHR | Agamous Like Mads Box Protein Agl80_Related | ✓ |
| **Glyma.20G117100** | 9615 | AT | Glutamine tRNA ligase | ✓ |
| **Glyma.20G152800** | 1844 | Pfam | Protein Kinase Domain (Pkinase) | ✓ |
| **Glyma.20G163500** | 1801 | PTHR | ABC Transporter G Family Member 4_Related | ✓ |
| **Glyma.20G194200** | 3709 | AT | Bis(5' Nucleosyl) Tetraphosphatase (Asymmetric | ✓ |

**Supplementary Table 4.** PCR-based validation of SVs called on the basis WGS data.

| **#** | **ID** | **CHR.** | **SV size (bp)** | **Frequency** | **PCR** |
| --- | --- | --- | --- | --- | --- |
| **1** | TR-intra | Chr05 | 707 | 0.52 | ✓ |
| **2** | TR-intra | Chr05 | 609 | 0.56 | ✓ |
| **3** | TR-inter | Chr07 | 586 | 0.03 | × |
| **4** | TR-inter | Chr10 | 321 | 0.47 | ✓ |
| **5** | TR-inter | Chr11 | 6,908 | 1 | ✓ |
| **6** | TR-inter | Chr05 | 373 | 1 | ✓ |
| **7** | TR-inter | Chr08 | 16,365 | 1 | ✓ |
| **8** | TR-inter | Chr10 | 6,279 | 0.07 | × |
| **9** | TR-inter | Chr13 | 170 | 0.49 | ✓ |
| **10** | TR-inter | Chr14 | 391 | 1 | ✓ |
| **11** | TR-inter | Chr15 | 10,123 | 0.44 | ✓ |
| **12** | TR-inter | Chr18 | 6,223 | 0.07 | × |
| **13** | TR-inter | Chr19 | 9,394 | 0.04 | × |
| **14** | TR-inter | Chr10 | 13,336 | 0.68 | ✓ |
| **15** | TR-inter | Chr13 | 224 | 0.31 | ✓ |
| **16** | TR-inter | Chr04 | 14,777 | 0.72 | ✓ |
| **17** | TR-inter | Chr06 | 8,354 | 0.66 | ✓ |
| **18** | TR-inter | Chr13 | 639 | 0.42 | ✓ |
| **19** | TR-inter | Chr15 | 594 | 0.87 | ✓ |
| **20** | TR-inter | Chr01 | 2,653,756 | 0.03 | × |
| **21** | INS | Chr01 | 43,758 | 0.56 | ✓ |
| **22** | INS | Chr02 | 12,374 | 0.25 | ✓ |
| **23** | INS | Chr20 | 6,238 | 0.31 | ✓ |
| **24** | INV | Chr13 | 83 | 0.41 | ✓ |
| **25** | INV | Chr07 | 214 | 0.44 | ✓ |
| **26** | INV | Chr10 | 251 | 0.21 | ✓ |
| **27** | INV | Chr11 | 40,657 | 0.05 | × |
| **28** | INV | Chr12 | 2,130 | 0.97 | ✓ |
| **29** | INV | Chr13 | 39,839 | 0.21 | ✓ |
| **30** | INV | Chr17 | 2,134,493 | 0.04 | × |
| **31** | INV | Chr17 | 5,729 | 0.93 | ✓ |
| **32** | INV | Chr19 | 2,130 | 0.63 | ✓ |
| **33** | INV | Chr15 | 36 | 0.29 | ✓ |
| **34** | INV | Chr19 | 2,221 | 0.16 | ✓ |
| **35** | INV | Chr06 | 136,631 | 0.04 | × |
| **36** | INV | Chr06 | 3,403 | 0.31 | ✓ |
| **37** | INV | Chr13 | 39,839 | 0.21 | ✓ |
| **38** | INV | Chr19 | 2,353,523 | 0.59 | × |
| **39** | DEL | Chr01 | 1,644 | 0.68 | ✓ |
| **40** | DEL | Chr19 | 15,502 | 0.14 | ✓ |

**Supplementary Table 5.** Concordance of WGS-based genotyping and PCR-based genotyping results for a deletion in *E3* gene and an insertion in *E4* gene.

| **#** | **ID** | **Sample Name** | ***E4* WGS genotype** | **PCR**  (✓or x) | ***E3* WGS genotype** | **PCR**  (✓or x) |
| --- | --- | --- | --- | --- | --- | --- |
| **1** | CAD_1001 | OAC Bayfield | E4 | ✓ | E3 | ✓ |
| **2** | CAD_1002 | OAC Prudence | e4-SORE-1 | ✓ | e3-tr | ✓ |
| **3** | CAD_1003 | OAC Wallace | E4 | ✓ | E3 | ✓ |
| **4** | CAD_1004 | AC Orford | E4 | ✓ | e3-tr | ✓ |
| **5** | CAD_1005 | Gaillard | E4 | ✓ | E3 | ✓ |
| **7** | CAD_1007 | AC Proteina | E4 | ✓ | E3 | ✓ |
| **8** | CAD_1008 | Mandarin | E4 | ✓ | e3-tr | ✓ |
| **9** | CAD_1009 | Evans | E4 | ✓ | E3 | ✓ |
| **10** | CAD_1010 | Maple Presto | e4-SORE-1 | ✓ | e3-tr | ✓ |
| **11** | CAD_1011 | OAC Ayton | E4 | ✓ | E3 | ✓ |
| **12** | CAD_1012 | OAC Kent | E4 | ✓ | e3-tr | ✓ |
| **14** | CAD_1014 | Dares | E4 | ✓ | E3 | ✓ |
| **15** | CAD_1015 | OAC Lakeview | E4 | ✓ | e3-tr | ✓ |
| **16** | CAD_1016 | OAC Morris | E4 | ✓ | E3 | ✓ |
| **17** | CAD_1017 | Maple Glen | E4 | ✓ | E3 | ✓ |
| **18** | CAD_1018 | Alta | e4-SORE-1 | ✓ | e3-tr | ✓ |
| **19** | CAD_1019 | OAC Champion | E4 | ✓ | E3 | ✓ |
| **20** | CAD_1020 | OT94-47 | e4-SORE-1 | ✓ | e3-tr | ✓ |
| **22** | CAD_1022 | OAC Pétrel | E4 | ✓ | e3-tr | ✓ |
| **49** | CAD_1049 | OAC Oxford | E4 | ✓ | E3 | ✓ |
| **50** | CAD_1050 | QS4004P.4J | E4 | ✓ | e3-tr | ✓ |
| **51** | CAD_1051 | Altesse | E4 | ✓ | E3 | ✓ |
| **53** | CAD_1053 | OAC Ginty | E4 | ✓ | E3 | ✓ |
| **55** | CAD_1055 | OAC Clinton | E4 | ✓ | E3 | ✓ |
| **56** | CAD_1056 | OAC Drayton | E4 | ✓ | E3 | ✓ |
| **57** | CAD_1057 | QS5055.43G | E4 | ✓ | e3-tr | ✓ |
| **64** | CAD_1064 | OAC Carman | E4 | ✓ | e3-tr | ✓ |
| **65** | CAD_1065 | OAC Madoc | E4 | ✓ | E3 | ✓ |
| **66** | CAD_1066 | QS4067P.17j | E4 | ✓ | E3 | ✓ |
| **67** | CAD_1067 | QS5085.8Bp | E4 | ✓ | e3-tr | ✓ |
| **70** | CAD_1070 | QS5091.50j | E4 | ✓ | E3 | ✓ |
| **71** | CAD_1071 | OAC Gretna | E4 | ✓ | E3 | ✓ |
| **72** | CAD_1072 | DH618(OAC06-18) | E4 | ✓ | E3 | ✓ |
| **73** | CAD_1073 | Bloomfield | E4 | ✓ | e3-tr | ✓ |
| **74** | CAD_1074 | OAC Stratford | E4 | ✓ | e3-tr | ✓ |
| **77** | CAD_1077 | AC 2001 | hetero | ✓ | e3-tr | ✓ |
| **78** | CAD_1078 | Kamichis | E4 | ✓ | E3 | ✓ |
| **79** | CAD_1079 | AC Proteus | E4 | ✓ | E3 | ✓ |
| **81** | CAD_1081 | Maple Amber | E4 | ✓ | e3-tr | ✓ |
| **83** | CAD_1083 | AC Bravor | E4 | ✓ | E3 | ✓ |
| **87** | CAD_1087 | Roland | E4 | ✓ | E3 | ✓ |
| **90** | CAD_1090 | 9004 | E4 | ✓ | e3-tr | ✓ |
| **91** | CAD_1091 | Heather | e4-SORE-1 | ✓ | e3-tr | ✓ |
| **92** | CAD_1092 | Maple Isle | e4-SORE-1 | ✓ | e3-tr | ✓ |
| **93** | CAD_1093 | 9063 | e4-SORE-1 | ✓ | e3-tr | ✓ |
| **95** | CAD_1095 | Jari | E4 | ✓ | E3 | ✓ |
| **100** | CAD_1100 | Dundas | E4 | ✓ | E3 | ✓ |
| **101** | CAD_1101 | Maple Donovan | e4-SORE-1 | ✓ | e3-tr | ✓ |
| **102** | CAD_1102 | Toki | e4-SORE-1 | ✓ | E3 | ✓ |
| **103** | CAD_1103 | Katrina | E4 | ✓ | E3 | ✓ |

**Supplementary Table 6**. Comparison of the contributions of this and previous large-scale studies to the characterization of both nucleotide and structural variation in soybean.

|  | Nucleotide variants | | | | Structural variants | | | | | | |
| --- | --- | --- | --- | --- | --- | --- | --- | --- | --- | --- | --- |
| Study | **SNPs** | **MNPs** | **Indel** | **Total** | **DEL** | **INS** | **DUP** | **INV** | **CNV** | **TRANS** | **Total** |
| Valliyodan et al. ‎2016^1^† | 10,417,285 | ND | 745,814 | 11,163,099 | 7,902* | | ND | ND | ND | ND | 7,902 |
| dos Santos et al. ‎2016^2^ | 5,835,185 | ND | 1,329,844 | 7,165,029 | 1,093 | | ND | ND | ND | ND | 1,093 |
| Zhou et al. 2015^3^ | 3,702,015 | ND | 325,720 | 4,027,735 | 6,388 | ND | ND | ND | 1,614 | ND | 8,002 |
| Anderson et al. ‎2014^4^‡ | ND | ND | ND | ND | 1,550 | | ND | ND | ND | ND | 1,550 |
| Lam et al. 2010^5^ | 4,127,942 | ND | 186,177 | 4,314,119 | ND | ND | ND | ND | ND | ND | ND |
| This study§ | **4,071,378** | **284,836** | **642,015** | **4,998,229** | **63,556** | **16,442** | **2,865** | **4,221** | **1,435** | **3313** | **91,832** |

**DEL**: deletion; **INS**; insertion; **DUP**: duplication; **INV**: inversion; **CNV**: copy-number variation; **TRANS**: translocation; **SNP**: single-nucleotide polymorphism; **MNP**: multiple nucleotide polymorphism; **Indel**: small insertion/deletion; **ND**: not determined

**†** Variant calling on a mixed population of *G. max* and *G. soja*; *Reported simply as presence/absence variants (PAVs); **‡** Structural variants detected using a CGH approach; **§** This is the only study which detected SV breakpoints at bp resolution and the only study which conducted variant validation tests.

1. Valliyodan B, Qiu D, Patil G, Zeng P, Huang J, et al. Landscape of genomic diversity and trait discovery in soybean. Scientific Reports. 2016; doi:10.1038/srep23598
2. dos Santos JVM, Valliyodan B, Joshi T, Khan SM, Liu Y, et al. Evaluation of genetic variation among Brazilian soybean cultivars through genome resequencing. BMC Genomics. 2016; doi:10.1186/s12864-016-2431-x
3. Zhou Z, Jiang Y, Wang Z, Gou Z, Lyu J, et al. Resequencing 302 wild and cultivated accessions identifies genes related to domestication and improvement in soybean. Nature Biotechnology. 2015; 33, 408–414. doi:10.1038/nbt.3096
4. Anderson JE, Kantar MB, Kono TY, Fu TY, Stec F, et al. A Roadmap for Functional Structural Variants in the Soybean Genome. G3. 2014;4(7):1307-1318. doi:10.1534/g3.114.011551.
5. Lam HM, Xu X, Liu X, Chen WB, Yang GH, et al. Resequencing of 31 wild and cultivated soybean genomes identifies patterns of genetic diversity and selection. Nat Genet. 2010; 42: 1053-1059. 10.1038/ng.715.
